# Photothermal–Immunomodulatory Hydrogel Reinforced by Ti_3_C_2_/ZnAl‐LDH Nanoplatform for Eradicating MRSA and Promoting Diabetic Wound Healing

**DOI:** 10.1002/advs.202517577

**Published:** 2026-01-20

**Authors:** Qiang Shi, Renliang Zhao, Jia Wang, Zhenchen Xiong, Xiangtian Deng, Cheng Zheng, Wenzhi Zhang

**Affiliations:** ^1^ Department of orthopaedics The First Affiliated Hospital of USTC Division of Life Sciences and Medicine University of Science and Technology of China Hefei Anhui P. R. China; ^2^ Orthopedics Research Institute Department of Orthopedics West China Hospital Sichuan University Chengdu P. R. China; ^3^ Trauma Medical Center Department of Orthopedics Surgery West China Hospital Sichuan University Chengdu P. R. China; ^4^ National Engineering Research Center for Biomaterials Sichuan University Chengdu P. R. China

**Keywords:** biomimetic NETs, hydrogel, immune regulation, MRSA, photothermal therapy

## Abstract

MRSA infections give rise to chronic cutaneous and advance into profound tissue invasions, including osteomyelitis and sepsis—conditions for which existing clinical interventions offer only limited efficacy. The utility of broad‐spectrum antibiotics has become increasingly compromised by escalating drug resistance, inadequate tissue targeting, and deleterious side effects. Neutrophil extracellular traps (NETs) have emerged as a compelling immunotherapeutic avenue for eradicating multidrug‐resistant pathogens. Inspired by the bactericidal and immunoregulatory functions of NETs, we developed a photothermal–immunomodulatory hydrogel reinforced by a Ti_3_C_2_/ZnAl‐LDH nanoplatform to mimic NETs‐like antimicrobial defense while overcoming their inherent limitations. Hollow photothermal nanoparticles were fabricated from 2D Ti_3_C_2_ and ZnAl‐LDH (TiZ) nanosheets and infused with chlorogenic acid, forming CTiZ. These were cloaked in membranes derived from apoptotic neutrophils to create a NETs‐mimetic functional bacterial trapper (CTiZM), which was then encapsulated within a thermoresponsive dopa‐poly(N‐isopropylacrylamide) hydrogel (dopa‐PNIPAm, DP), yielding a composite hydrogel system designated CTiZM@DP. This sophisticated platform amplifies antibacterial potency through effective microbial entrapment and the generation of reactive oxygen species, while simultaneously orchestrating the polarization of macrophages toward a reparative M2 phenotype under the influence of gentle photothermal stimulation. Collectively, this integrative strategy heralds a promising therapeutic modality for the eradication of multidrug‐resistant infections and the enhancement of wound healing in individuals with diabetes.

## Graphical abstract

A NETs‐inspired Ti_3_C_2_/LDH hydrogel integrates trapping, ROS boosting, and M2‐immunomodulation for MRSA‐infected wound repair.

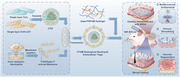

## 1 Introduction

Methicillin‐resistant Staphylococcus aureus (MRSA) is characterized by high invasiveness, elevated mortality, and extensive multidrug resistance [1]. MRSA‐associated wounds typically exhibit persistent inflammation, impaired angiogenesis, and delayed tissue regeneration. Chronic cutaneous lesions caused by MRSA present formidable clinical challenges due to robust biofilm formation, multidrug resistance, and the inability of conventional antibiotics to maintain effective local concentrations [2, 3, 4]. Although emerging therapeutic approaches—such as bacteriophage therapy and stimuli‐responsive biomaterials—have provided new avenues, they remain limited by insufficient capability to overcome resistance, poor tissue‐targeting efficiency, and singular antibacterial mechanisms. Recent evidence indicates that neutrophil extracellular traps (NETs) can physically immobilize bacteria through their DNA meshwork, thereby restricting microbial dissemination, while exerting direct cytotoxic effects via reactive oxygen species (ROS), histones, and antimicrobial peptides [5, 6]. NETs also promote immune‐cell recruitment and facilitate MRSA clearance, demonstrating substantial therapeutic potential in complex chronic wounds [7]. However, MRSA has evolved multiple strategies to evade NETs‐mediated killing, including secreting nucleases that degrade the DNA scaffold of NETs, producing staphyloxanthin to quench oxidative stress, suppressing NETs formation, and modifying its surface structures to reduce NETs' affinity. These mechanisms collectively sustain chronic infection and greatly increase treatment difficulty [8, 9]. To address these challenges, we propose the development of biomimetic NETs as an adjunct immunotherapeutic strategy to reinforce host defense against MRSA, particularly in diabetic and immunocompromised individuals. This engineered construct aims to recapitulate the functional characteristics of natural NETs by encapsulating nanoparticles within DNA–protein complexes or cell membrane structures to precisely mimic bacterial trapping and bactericidal activity [10]. Moreover, the integration of antimicrobial agents enables temporally controlled drug release, thereby reproducing the sustained antibacterial effect of natural NETs; in addition, conjugating DNA with antimicrobial proteins can further enhance antibacterial efficacy.

Incorporating biomimetic NETs into vesicular, liposomal, or nanoparticulate delivery platforms augments their structural integrity, enhances tissue targeting, and potentiates immune engagement, thus supporting multimodal therapeutic applications [11]. Yet, a central translational challenge lies in balancing immune stimulation with the imperative to mitigate collateral tissue injury. 2D materials have garnered substantial attention for antibacterial purposes. Reconfiguring these into 3D hollow nanostructures markedly expands their surface area and drug‐loading capabilities, impedes sheet aggregation, improves colloidal stability, and enables the convergence of diverse functionalities with stimulus‐responsive release dynamics. Such features endow these platforms with superior control over pharmacokinetics, targeted delivery, and synergistic immuno‐antibacterial actions [12, 13]. Layered nanomaterials, notably layered double hydroxides (LDHs), demonstrate exceptional bacterial binding affinity and photothermal conversion efficacy, rendering them ideal for noninvasive bacterial eradication under near‐infrared (NIR) irradiation [14]. Building upon this foundation, chlorogenic acid (CGA) was encapsulated within the hollow nanostructures to augment bacterial adhesion and, following macrophage uptake, elicit innate immune activation for successive pathogen clearance and tissue regeneration [14]. Surface modification with neutrophil‐derived membranes further endowed the particles with bacterial tropism via receptor‐mediated targeting and immune activation through exposed “eat‐me” signals such as phosphatidylserine, enabling both persistent and finely tuned immunomodulation [15, 16].

**SCHEME 1:**
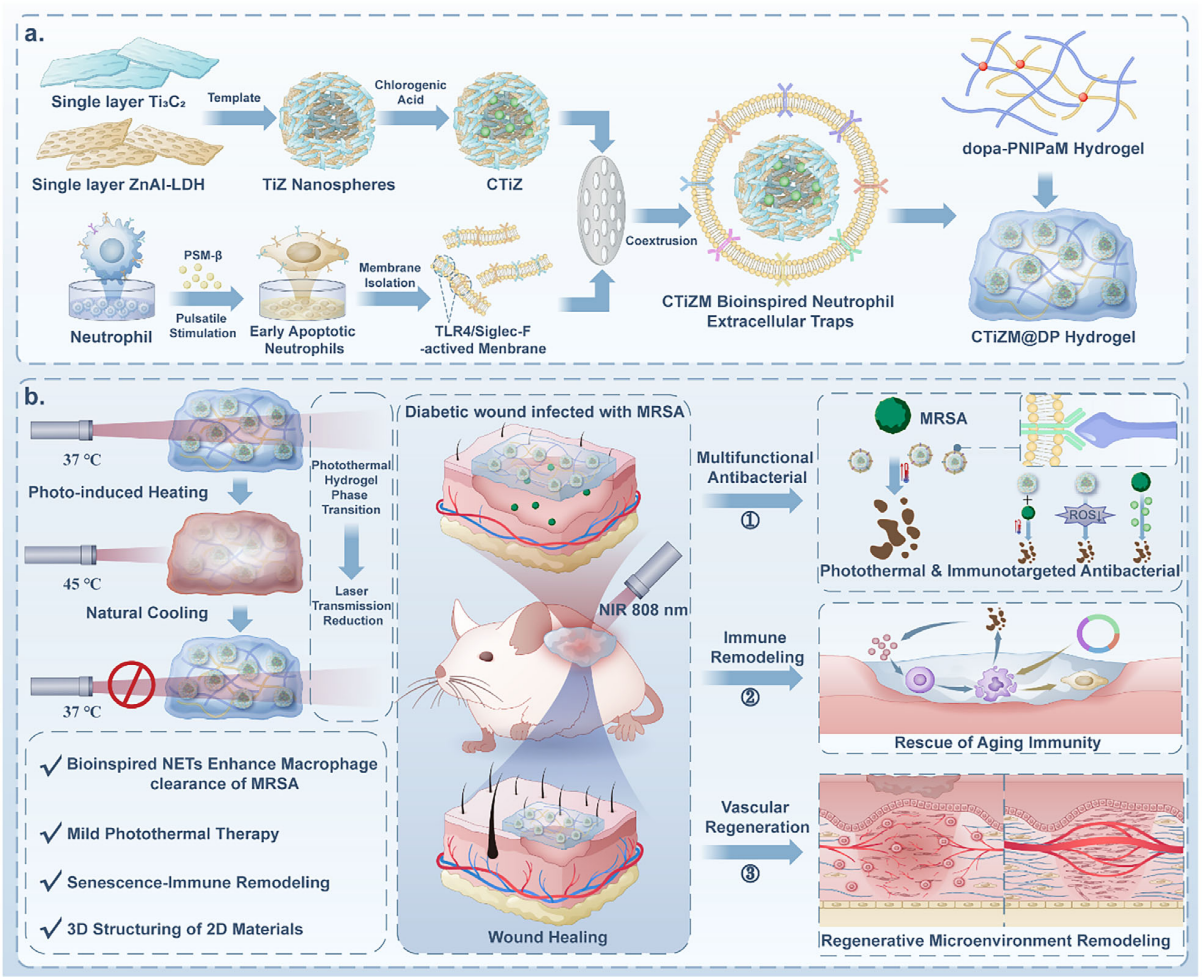
Schematic illustration of the synthesis of CTiZM@DP bionic NETs and their treatment in diabetic MRSA‐infected wounds.

In this work, we engineered hollow nanoparticles via a templated self‐assembly approach using 2D Ti_3_C_2_ and ZnAl‐LDH nanosheets, which were subsequently loaded with CGA to yield CGA@Ti_3_C_2_ZnAl‐LDH(CTiZ). Apoptotic neutrophil membranes were then co‐extruded with CTiZ to form biomimetic NET‐like nanoparticles (CTiZM). These nanostructures exhibited pronounced photothermal responsiveness under NIR illumination. The inclusion of bacterial‐specific ligands (TLR4, Siglec‐F) on the membrane surface enhanced pathogen binding, while phosphatidylserine exposure facilitated macrophage recognition and phagocytic activation. To enable localized and controlled therapeutic delivery, CTiZM was further embedded into a thermoresponsive dopa‐PNIPAm (DP) hydrogel, serving as an injectable wound dressing. The hydrogel's phase transition modulated NIR transmittance, sustaining CTiZM's mild photothermal functionality. Both in vitro and in vivo studies, including a diabetic rat model of MRSA‐infected wounds, confirmed the system's potent antimicrobial efficacy, immune activation, and wound‐healing capabilities (Scheme 1). In summary, this platform presents a sophisticated therapeutic paradigm for managing chronic MRSA‐infected diabetic wounds and holds considerable promise for the precise treatment of infections involving both resistant and susceptible bacterial strains in clinical practice.

## 2 Results and Discussion

### 2.1 Preparation and Characterization of CTiZM Bioinspired NETs

Neutrophil extracellular traps (NETs) entrap pathogens and orchestrate immunocide through a complex, multiscale architecture—comprising the reticular framework of DNA filaments and the functional moieties of associated proteins. 2D materials, such as graphene, MXenes, and layered double hydroxides, emerge as ideal biomimetic platforms for NETs due to their high specific surface area, tunable surface charge, and abundant sites for multifunctional modification. By engineering these 2D materials into 3D architectures, one can emulate the porous, multidimensional topology characteristic of NETs—endowing the mimetic constructs with histone‐like charge distributions and pathogen‐capturing capabilities. Furthermore, the inherent multifunctionality of these materials enables dynamic responsiveness and synergistic action, markedly augmenting their antimicrobial efficacy and immunoregulatory potential. Ti_3_C_2_ has been extensively employed in antimicrobial and tissue regeneration applications owing to its remarkable photothermal properties. Recent studies have revealed that structural modification of Ti_3_C_2_ with dihydroperoxides—endowed with innate bacterial adsorption capabilities—can synergistically amplify its bacterial entrapment efficiency and bactericidal potential [17].

**FIGURE 1:**
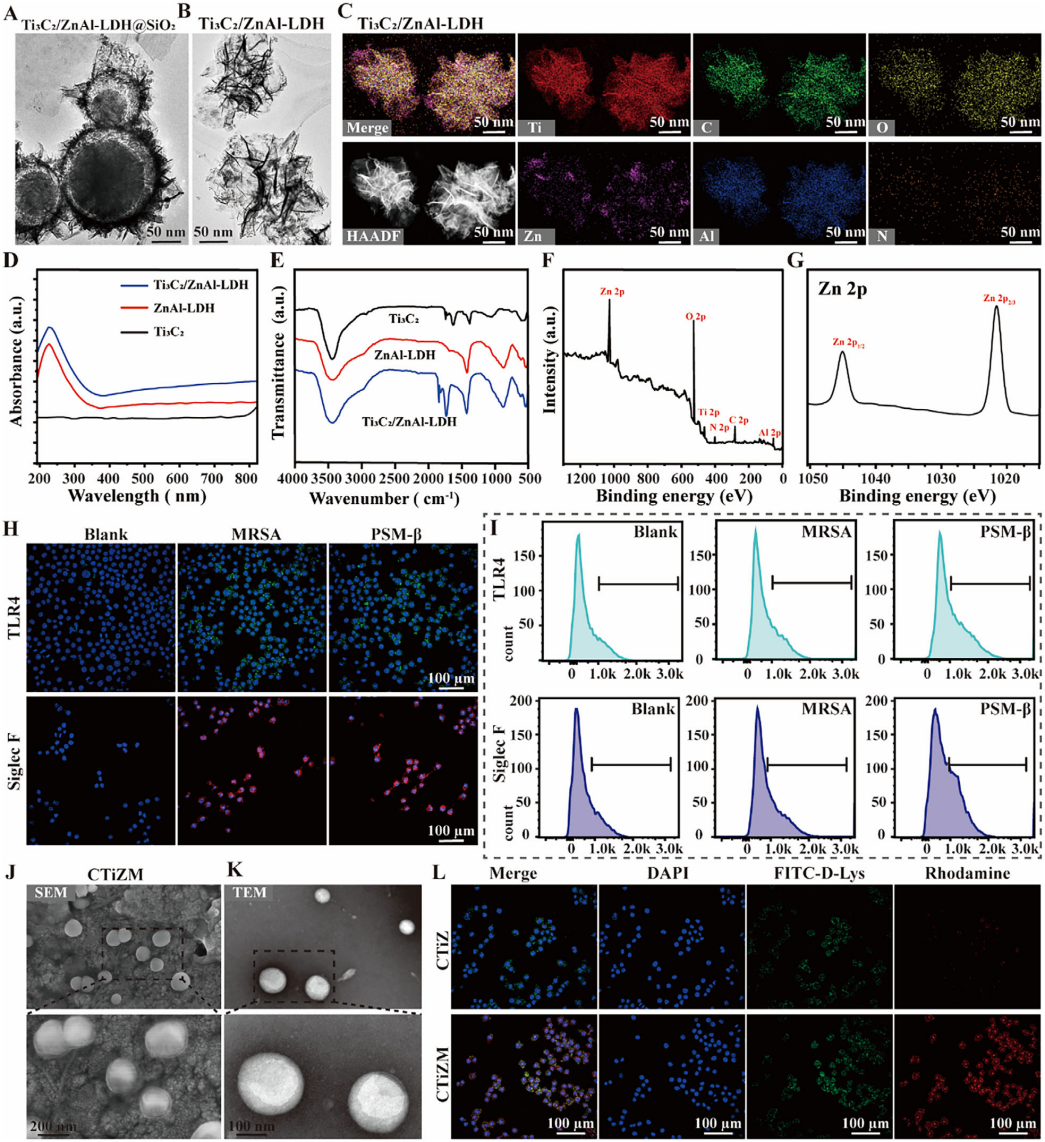
(A) TEM images of Ti3C2 / ZnAl‐LDH@SiO_2_ nanoparticles (TiZ@SiO_2_) prepared by the template method. (B) TEM of TiZ nanoparticles. (C) mapping images of TiZ nanoparticles. (D) Absorbance of TiZ nanoparticles. (E) FTIR of TiZ nanoparticles; (F) XPS of TiZ nanoparticles. (G) XPS analysis of elemental Zn. (H) Expression of TLR4 and Siglec‐F on the surface of neutrophils after MRSA and PSM‐β interventions, respectively. (I) Flow analysis of the fluorescence intensity of TLR4 and Siglec‐F on the surface of neutrophils. (J) SEM of TiZ nanoparticles after membrane encapsulation (termed as TiZM). (K) TEM of TiZM nanoparticles. (L) Fluorescence co‐localization of TiZM nanoparticles and bacteria within macrophages using, respectively, the Rhodamine‐labeled TiZM, FITC‐D‐Lys labeled bacteria, and DAPI‐labeled nucleus.

In this study, we utilized 3D nanoparticles constructed by the template method by combining Ti_3_C_2_ and ZnAl‐LDH. Transmission electron microscopy (TEM) can observe that its surface consists of lamellar structures, and the multilayered lamellar structure constitutes the hollow nanoparticles (Figure 1A). TEM was carried out to observe the particle size and internal structure, and it was found that the nanoparticles consisted of disordered single‐layer 2D materials forming a nanoflake‐like structure, from which the internal hollow structure could be observed (Figure 1B). The elements of the nanoparticles were further analyzed, and it was found that the elements within the nanoparticles were well‐distributed, and the 2D nanosheets of Ti_3_C_2_ and ZnAl‐LDH were randomly and evenly distributed in the 3D nanospheres (Figure 1C). The Ultraviolet–visible (UV) absorption spectrum shows a higher absorption peak for ZnAl‐LDH at lower wavelengths, while Ti_3_C_2_ exhibits no absorption peak in this region (Figure 1D). The analysis of Ti_3_C_2_ using FTIR revealed that the peaks located at about 3459, 1643, and 559 cm‐^1^ were attributed to the characteristic peaks of−OH, C═O, and Ti─O groups, respectively, whereas the presence of ZnAl‐LDH in the ZnAl‐LDH layer was observed at about 581 and 428 cm‐^1^ on ZnAl‐LDH. The presence of characteristic peaks of Zn─OH and Zn─O groups, all of their characteristic peaks, and characteristic peaks of Ti─O groups indicates that Ti_3_C_2_ / ZnAl‐LDH nanoparticles were successfully prepared (Figure 1E). The functional groups of Ti_3_C_2_ / ZnAl‐LDH nanoparticles were examined by XPS, and the results showed that all the major Zn 2p peaks were located near 1044.7, and the characteristic peaks of Ti 2p were mainly near 453.8. Further evaluation of the Zn 2p peaks in the nanoparticles revealed that the major Zn 2p3/2 and Zn 2p peaks are located around 1044.7. Zn 2p3/2 and Zn 2p1/2 peaks located at 1044.7 and 1021.6 eV, respectively (Figure 1F,G; Figure S1A,B). TLR4 and Siglec‐F serve as critical molecular checkpoints in the recognition and phagocytosis of MRSA by neutrophils. Accumulating evidence suggests that elevated expression of Siglec‐F facilitates MRSA detection but may also contribute to immunosuppression following acute infection—potentially representing a mechanism of immune evasion by MRSA. In this study, we employed pulsed, low‐concentration stimulation of neutrophils using PSM‐β, a key virulence factor of MRSA, and observed a marked upregulation of both TLR4 and Siglec‐F on the neutrophil surface (Figure 1H,I), accompanied by signs of early‐stage apoptosis (Figure S2). Subsequently, we harvested the membranes from neutrophils undergoing early apoptosis to encapsulate CTiZ nanoparticles, thereby fabricating biomimetic NETs, denoted as CTiZM. Scanning and transmission electron microscopy revealed a distinct membrane‐like architecture on the surface of CTiZM nanoparticles, with TEM clearly distinguishing the outer membrane layer from the inner nanoparticulate core, forming a spherical, membrane‐wrapped morphology (Figure 1J,K).  Dynamic light scattering (DLS) analysis validated that the particle size of the prepared CTiZM was mostly concentrated at 155 nm (Figure S3). In addition, the alteration of surface zeta potentials of nanocomposites proved the successful modification in each step (Figure S4). To verify the successful coating of CTiZ nanoparticles with early apoptotic neutrophil membrane (NM), fluorescence microscopy was employed to visualize their co‐localization. As illustrated in the figures, rhodamine B isothiocyanate (RBITC)‐labeled CTiZ nanoparticles and Annexin V‐fluorescein isothiocyanate (Annexin V‐FITC)‐labeled NM exhibited a consistent spatial distribution, indicating efficient membrane coating of CTiZ by NM (Figure S5). Figure S6 presents the in vitro release profiles of chlorogenic acid (CGA) from CTiZM under NIR irradiation and non‐irradiation conditions. In the absence of NIR irradiation, CGA displayed a sustained‐release characteristic, with the cumulative release reaching only approximately 20% after 24 h. In contrast, NIR irradiation significantly accelerated drug release: the release percentage rapidly increased to around 55% within the first 4 h, and the cumulative release reached approximately 85% at 24 h. These results clearly demonstrate that NIR irradiation significantly enhances CGA release, highlighting the excellent photo‐responsive controlled‐release capability of CTiZM (Figure S6). FITC‐D‐Lys was employed to fluorescently label the bacteria, rhodamine red for the bionic NETs, and DAPI for macrophages. Upon co‐culturing the three components, we observed substantial bacterial uptake within macrophages, with CTiZM markedly enhancing the macrophages’ capacity for bacterial capture and phagocytosis (Figure 1L). Finally, CTiZM was exposed to three different aqueous environments (PBS, α‐MEM, and α‐MEM + 10% FBS) for 7 days to evaluate changes in particle size over time, further demonstrating the remarkable physiological stability of CTiZM (Figure S7).

### 2.2 Preparation of CTiZM@DP Photosensitive Hydrogel, Bioactivity Assay in Vitro and Evaluation of Antimicrobial Properties

NETs can remove bacteria by trapping them and triggering a ROS storm, which is one of the key mechanisms for their immuno‐antimicrobial effects. Bionic NETs are capable of rapidly generating ROS storms through a non‐invasive photothermal effect to efficiently clear MRSA locally. However, its accompanying overheating effect may cause damage to the surrounding normal tissues, a shortcoming that greatly limits its application in the clinic. Researchers found that the development of materials with a mild photothermal effect could achieve an effective antimicrobial effect at conditions below 45°C. The use of mild thermal stimulation, in collaboration with physical and immune therapeutic modalities, to develop new antibacterial mechanisms targeting bacterial membrane permeability and enzyme activity is of vital significance for enhancing antibacterial effects, reducing toxic side effects, and improving the possibility of clinical translation.

**FIGURE 2:**
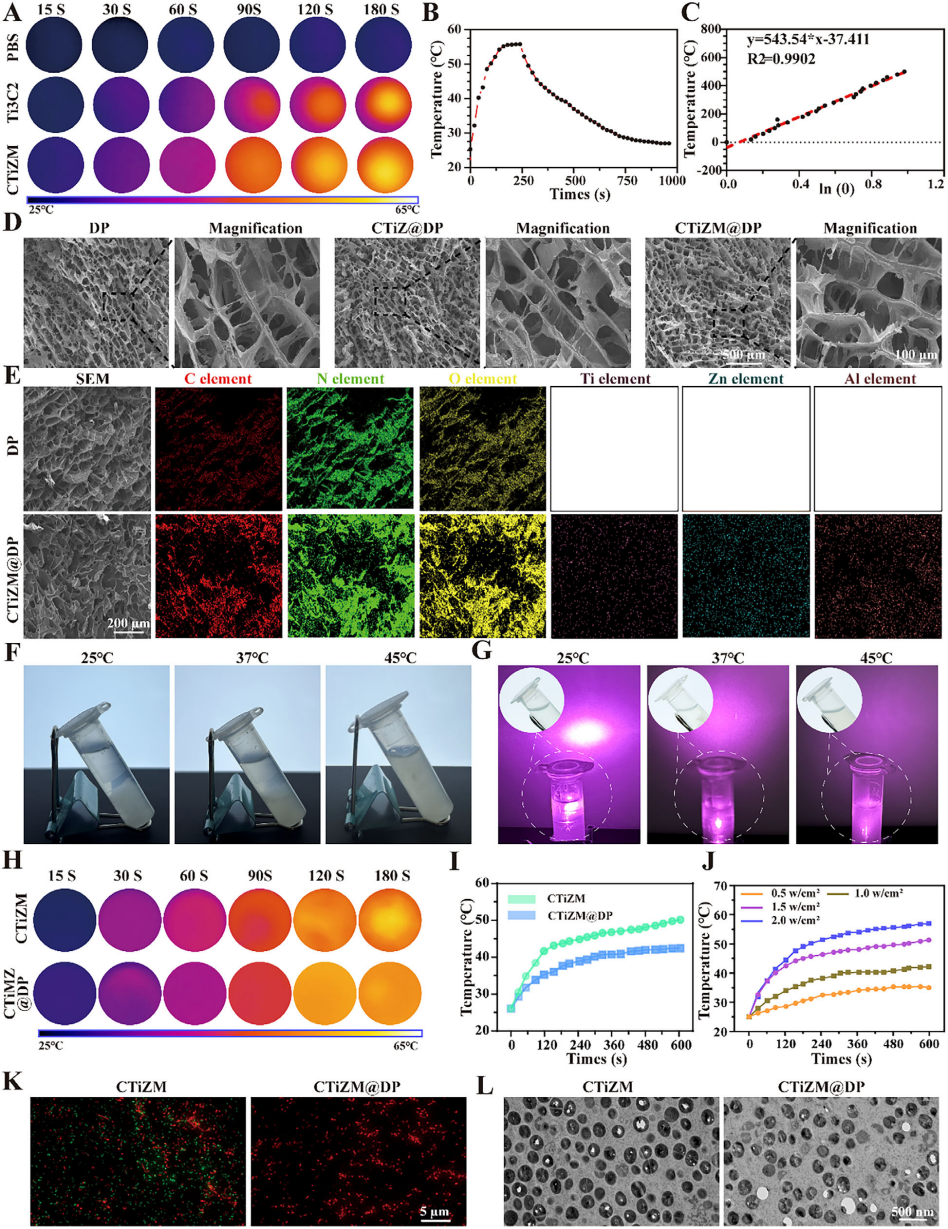
(A) In vitro temperature change images of CTiZM nanoparticles under light.(B) Cooling curves of CTiZM nanoparticles photothermally. (C) Photothermal conversion curves of CTiZM nanoparticles. (D) Scanning electron microscope images of dopa‐PNIPAm (DP) hydrogel; (E) SEM and Mapping elemental analysis of CTiZM@DP hydrogel.(F) State and appearance of CTiZM@DP hydrogel under different temperatures. (G) NIR transmittance of CTiZM@DP hydrogel under different temperatures. (H) In vitro temperature change images of CTiZM@DP hydrogel under light. (I) Warming curves of CTiZM@DP hydrogel. (J) Warming curves of CTiZM@DP hydrogel at different powers. (K) In vitro bacterial viability and death staining evaluation of antimicrobial capacity of CTiZM@DP hydrogel. (L) Bacterial TEM of MRSA.

The photothermal behavior of CTiZM nanoparticles was first evaluated in vitro. Upon NIR irradiation, a time‐dependent increase in temperature was observed, reaching approximately 60°C within 180 s (Figure 2A). After 250 s of irradiation, the nanoparticles were allowed to cool naturally, producing a smooth and continuous cooling curve (Figure 2B). Based on these heating and cooling profiles, the photothermal conversion efficiency was calculated, demonstrating that CTiZM nanoparticles possess a favorable photothermal conversion rate (Figure 2C). To achieve controlled mild‐temperature photothermal performance, CTiZM nanoparticles were subsequently incorporated into a DP hydrogel matrix. SEM imaging revealed that the DP hydrogel exhibited an irregular porous microstructure (Figure 2D). Following nanoparticle loading, SEM and elemental mapping confirmed that CTiZM nanoparticles were successfully integrated and uniformly dispersed throughout the hydrogel network (Figure 2E). Rheological analysis further showed that both the PNIPAM hydrogel and CTiZM@DP hydrogel displayed typical viscoelastic characteristics, with storage modulus (G′) consistently exceeding loss modulus (G″) across the tested frequency range. Notably, the CTiZM@DP hydrogel demonstrated significantly higher G′ and G″ values, indicating enhanced mechanical integrity (Figure S8).

The color change and phase transition of the CTiZM@DP composite hydrogel were examined under different temperatures. As the temperature increased, the hydrogel gradually crosslinked and darkened, accompanied by a significant reduction in NIR transmittance (Figure 2F,G). The photothermal characteristics of the hydrogel were further assessed using an infrared thermal imaging camera. Under continuous NIR irradiation, the temperature stabilized at approximately 45°C, indicating that the photosensitive hydrogel reliably maintained a mild photothermal effect (Figure 2H). Although higher NIR power led to an elevated temperature, all values remained below 55°C, and the photothermal output could be precisely modulated by adjusting the irradiation power (Figure 2I,J; Figure S9). To investigate the photothermal‐responsive release behavior, the release kinetics of CTiZM nanoparticles from the CTiZM@DP hydrogel were evaluated with or without NIR irradiation. As shown in Figure S10, spontaneous nanoparticle release was negligible under physiological conditions, with less than 10% released within 100 min. In contrast, 808 nm NIR irradiation triggered a rapid burst release, exceeding 80% within 80 min. This pronounced difference confirms the hydrogel's effective photothermal‐triggered release capability and its ability to achieve precise temporal control over nanoparticle delivery. Given the critical roles of 3T3‐L1 cells and HUVECs in wound repair, both cell types were used to assess the biological performance of the CTiZM hydrogels. CCK‐8 assays demonstrated that none of the hydrogel formulations exerted cytotoxicity during 1, 3, and 5 days of culture (Figure S11A,B). Live/Dead staining further revealed predominantly green fluorescence and progressive cell proliferation across all groups, regardless of NIR exposure, confirming excellent cytocompatibility (Figure S12A,B).

Finally, scratch assays were conducted to evaluate the influence of the hydrogels on cell migration. Images captured at 0 h and 24 h demonstrated that CTiZM under photothermal activation most prominently promoted the migration of both 3T3‐L1 cells and HUVECs (Figure S13A,B). Finally, to evaluate the antimicrobial capacity of bionic NETs composite gels with mild photothermal heat, the antimicrobial capacity was evaluated using live/dead bacteria staining and TEM at different powers, and it was found that good antimicrobial capacity was found for the CTiZM@DP gels (Figure 2K), and the death process of vacuolization and fragmentation of the MRSA interior could be observed by TEM (Figure 2L) and the antimicrobial capacity varied with photothermal power increased significantly. The antibacterial activities of bionic neutrophil extracellular trap (NETs)‐mimetic nanoparticles and composite hydrogel systems were further compared. In vitro antimicrobial assays further confirmed the potent bactericidal efficacy of CTiZM, as evidenced by a significant reduction in MRSA colony formation (Figure S14A,B). TEM imaging demonstrated clear bacterial destruction, likely attributable to the synergistic photothermal and physical antimicrobial properties inherent to the 2D material components of CTiZM (Figure S14C,D). Collectively, these findings underscore the superior MRSA‐trapping and bactericidal capabilities of CTiZM nanoparticles and highlight their potential to augment macrophage‐mediated bacterial clearance.

### 2.3 CTiZM@DP Hydrogel Targets Senescent Macrophages to Rescue Senescence and Remodel the Immune Ecological Niche

The prolonged presence of bacterial infection within chronic infected wounds in diabetes induces long‐term validation of the wounds, continuous stimulation of local macrophages by bacterial metabolites, which induces the initiation of the macrophage senescence program, reactive oxygen species damage to DNA leading to phenotypic alterations, decreased phagocytosis and bactericidal capacity, decreased antigen presentation function, and decreased secretion of inflammatory factors [18]. It has been shown that repair of damaged DNA in senescent cells by STAT6 can rescue macrophage senescence and damage and increase the antimicrobial capacity and healthy lifespan of macrophages [19]. In addition, it has been found that by removing excess ROS from the site of diabetic injury, the immune environment can be remodeled to enhance photothermal antimicrobial capacity while alleviating local inflammatory responses. This suggests that the senescent state of macrophages impairs their normal physiological functions and that improved senescence can better utilize their immunomodulatory abilities [20, 21].

**FIGURE 3:**
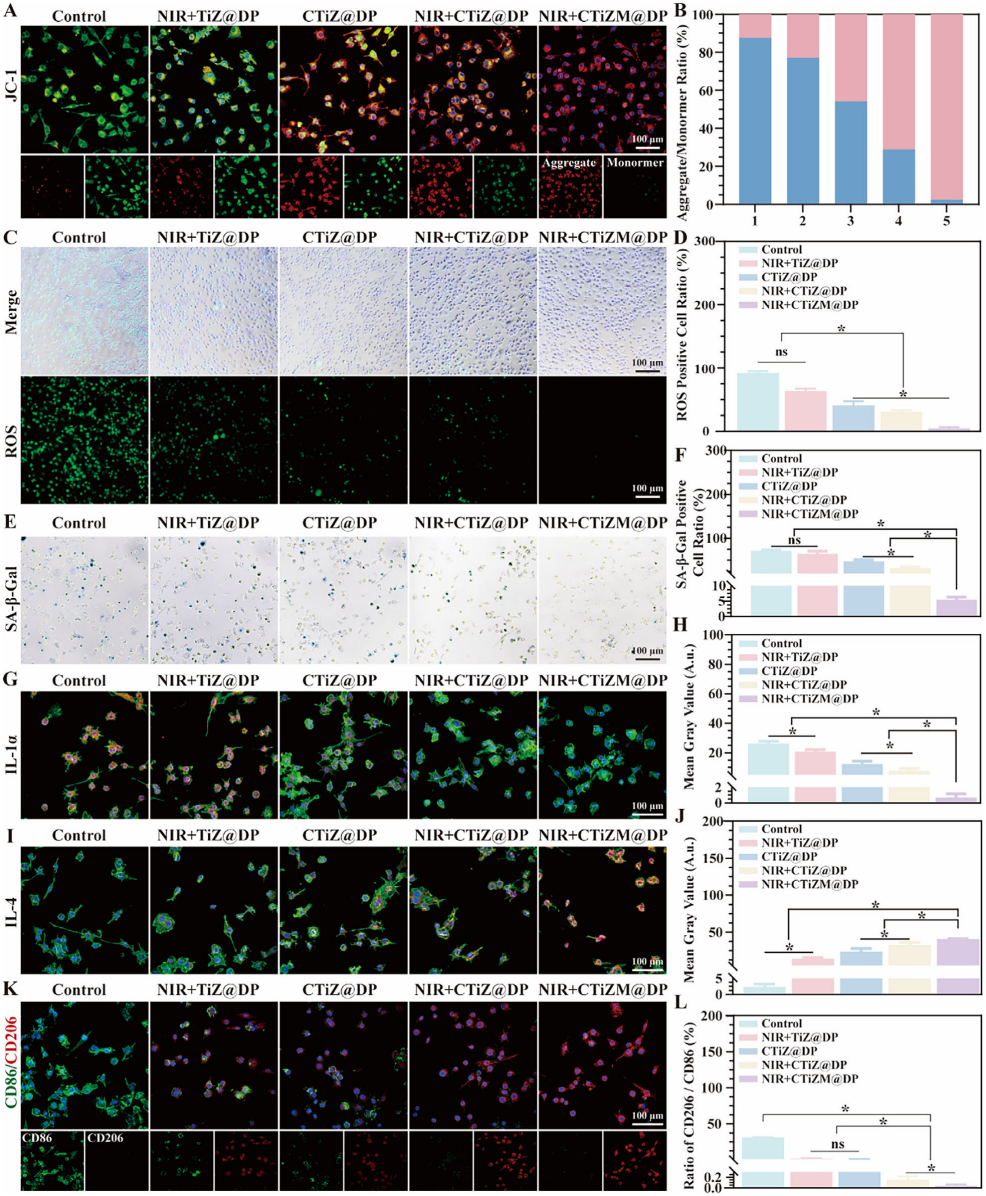
(A) JC‐1 assessment of mitochondrial membrane potential alteration in macrophages. (B) Statistical analysis of JC‐1 fluorescent probe assessment of multimers/monomers. (C) DCFH‐DA fluorescent probe for detection of intracellular ROS levels in macrophages. (D) statistical analysis of intracellular ROS fluorescence ratios in macrophages. (E) assessment of intracellular β‐galactosidase levels in macrophages. (F) β‐galactosidase‐positive macrophages statistical analysis. (G) Immunofluorescence detection of IL‐1α expression in co‐cultured macrophages, green fluorescence labeling Actin, red fluorescence labeling IL‐1α, and blue labeling of nuclei. (H) Statistical analysis of IL‐1α expression in macrophages. (I) Immunofluorescence detection of IL‐4 expression in co‐cultured macrophages, green fluorescence labeling of Actin, red fluorescence labeling of IL‐4, blue labeling of nuclei. (J) Statistical analysis of IL‐4 expression within macrophages. (K) Immunofluorescence identification of phenotypes within co‐cultured macrophages, green fluorescence labeling of CD86, red fluorescence labeling of CD206, and blue labeling of nuclei. (L) Statistical analysis of the CD86/CD206 ratio within macrophages. (Data represent independent experiments; all data are presented as mean ± SD with a sample size of n = 3; statistical analysis was performed using GraphPad Prism 9 software via one‐way analysis of variance (one‐way ANOVA); ns indicates not significant, ^*^ *p* < 0.05).

In this study, we evaluated macrophage status after MRSA stimulation and the ability of CTiZM@DP photoheated gel to modulate macrophage phenotype. Macrophage mitochondrial membrane potential was first evaluated, and the JC‐1 probe revealed a large amount of green fluorescence in MRSA‐stimulated macrophages, while CTiZM@DP Hydrogel significantly alleviated the extent of mitochondrial damage and restored the normal level of membrane potential in the macrophage (Figure 3A,B). Next, ROS levels within macrophages after MRSA stimulation were evaluated, and it was found that NIR‐irradiated CTiZM@DP Hydrogel significantly reduced the green fluorescence levels within macrophages, with statistically significant differences (Figure 3C,D). It was further evaluated whether MRSA‐stimulated ROS increase and mitochondrial damage led to macrophage senescence program initiation. The results showed that a large amount of green senescence, characterized by β‐galactosidase expression in macrophages after MRSA stimulation, while photothermal CTiZM@DP Hydrogel could significantly reduce the cellular senescence level with a statistically significant difference (Figure 3E,F). Macrophage immunomodulatory function was next evaluated by co‐culturing bacterially infected macrophages with normal macrophages, and it was found that macrophages induced large amounts of IL‐1α expression after MRSA stimulation, whereas photothermal CTiZM@DP Hydrogel reduced IL‐1α expression (Figure 3G,H), and increased IL‐4 expression, with a statistically significant difference noted (Figure 3I,J). Finally, the co‐cultured macrophage phenotypes were characterized, and it was found that photothermal CTiZM@DP Hydrogel induced M2‐type macrophage transformation and decreased M1 macrophage expression, with statistically significant differences (Figure 3K,L).

### 2.4 CTiZM@DP Photohydrothermal Gel Remodels Regenerative Microenvironment by Modulating Fibroblast Phenotype

In chronic wound repair, fibroblasts are the main cells that synthesize extracellular matrix (ECM) [22]. They gradually fill the wound defect area through components such as collagen, fibronectin, and elastic fibers, and dynamically regulate the ECM to achieve wound repair. However, in the continuous inflammatory process of chronic wounds, a large amount of reactive oxygen species (ROS) and inflammatory factors leads to the failure of fibroblasts to transform into myofibroblasts in time. The obstruction of their activity and phenotypic transformation can cause delayed wound repair or excessive fibrosis and pathological scar formation [23]. Studies have found that activating fibroblast phenotypic transformation through platelet‐derived growth factor can accelerate the wound repair process. Low‐intensity laser or ultrasound therapy can stimulate the function of fibroblasts. Through mechanical effects, it can promote fibroblast activation and also promote wound repair [24].

**FIGURE 4:**
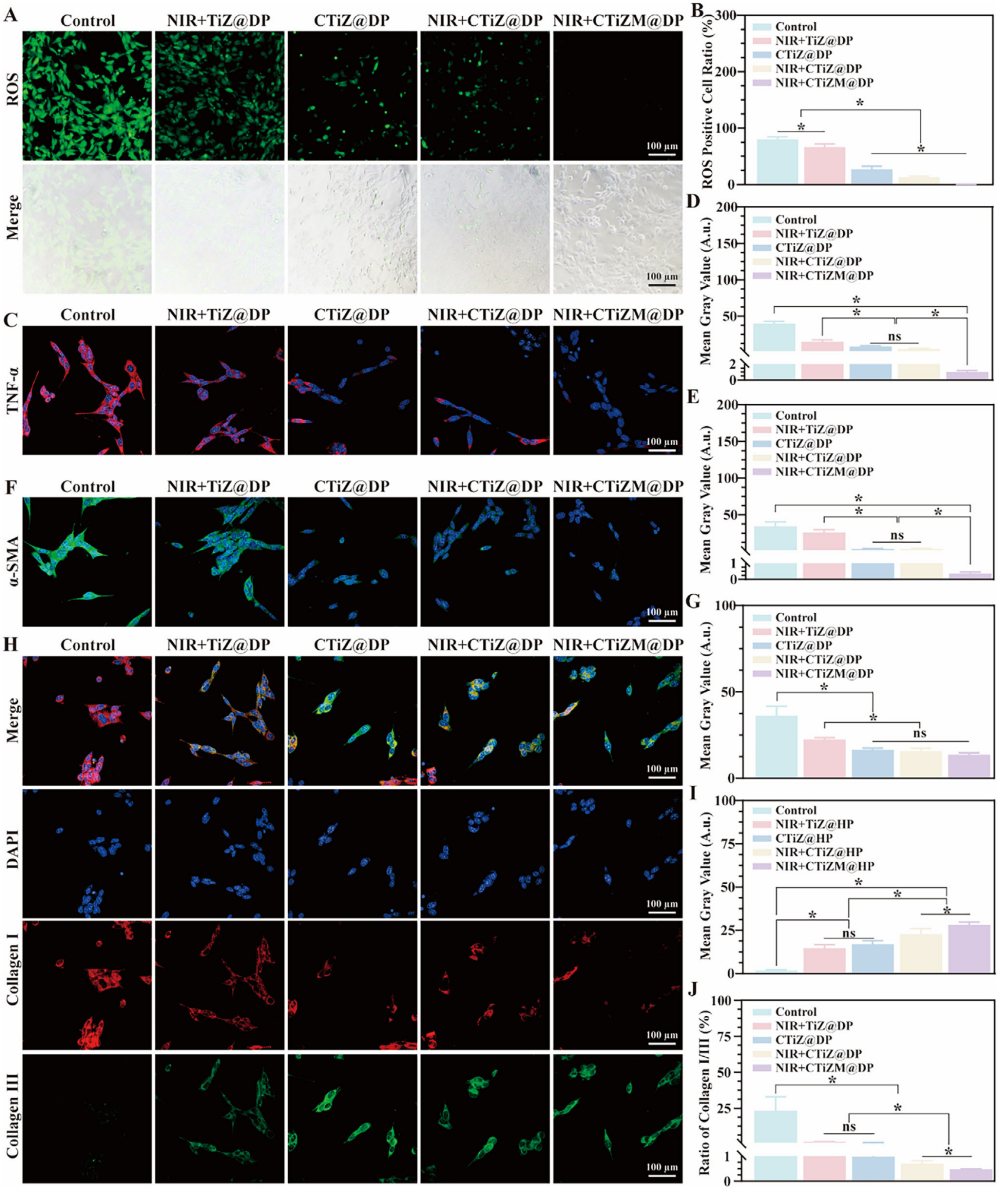
(A) Evaluation of ROS expression in fibroblasts. (B) Statistical analysis of ROS‐positive cells. (C) Analysis of TNF‐α expression in fibroblasts. (D) Statistical analysis of TNF‐α in fibroblasts. (E) Statistical analysis of α‐SMA expression in fibroblasts; (F) Evaluation of α‐SMA expression in fibroblasts. (G) Statistical analysis of type I collagen in fibroblasts. (H) Immunofluorescence evaluation of the expression of type I and type III collagen in fibroblasts. (I)Statistical analysis of type III collagen in fibroblasts. (J) Analysis of the ratio of type I/III collagen in fibroblasts. (Data represent independent experiments; all data are presented as mean ± SD with a sample size of n = 3; statistical analysis was performed using GraphPad Prism 9 software via one‐way analysis of variance (one‐way ANOVA); ns indicates not significant, ^*^ *p* < 0.05).

First, the state of fibroblasts in chronic wounds was evaluated. A co‐culture system was established to co‐culture MRSA‐infected macrophages and fibroblasts. The ROS level of fibroblasts in the co‐culture system was detected by DCFH‐DA green fluorescent probe. The results showed that the CTiZM@DP photothermal hydrogel could significantly reduce the excessive ROS in fibroblasts, and the results had statistical differences (Figure 4A,B). A high concentration of TNF‐α at the injury site may induce cells to produce excessive ROS, damage biological macromolecules such as DNA, proteins, and lipids in cells, and interfere with the normal metabolism and proliferation processes of cells. In chronic wounds related to chronic inflammatory diseases, a continuously high concentration of TNF‐α may inhibit the proliferation of fibroblasts and thus delay wound repair. We evaluated the level of TNF‐α in co‐cultured fibroblasts. The results showed that the CTiZM@DP photothermal hydrogel significantly reduced the level of TNF‐α in fibroblasts, and the statistical results were significantly different (Figure 4C,D). Further evaluation was conducted on the level of α‐SMA, which is crucial for fibroblast phenotypic transformation. The excessive expression of α‐SMA will lead to the generation of local excessive fibrosis. The results showed that the CTiZM@DP photothermal hydrogel significantly reduced the expression level of α‐SMA, and the statistical results were significantly different (Figure 4E,F). Fibroblasts participate in the wound repair process by secreting different types of collagen fibers. The collagen ratio determines the repair level and effect of the wound. The results found that infected macrophages will promote fibroblasts to secrete a large amount of type I collagen, which may lead to the generation of pathological scars. However, the CTiZM@DP photothermal hydrogel can reduce the expression of type I collagen and increase the proportion of type III collagen, making it more inclined to the state of normal skin healing. The statistical results are significantly different (Figure 4G,H,I,J).

### 2.5 In vivo Antimicrobial and Pro‐restorative Effects of CTiZM@DP Photosensitive Hydrogel

Chronic wound infection in diabetes mellitus is one of the main causes of non‐healing and deterioration of wounds [25]. Antimicrobial therapy can effectively kill bacteria on the surface of wounds and in the deeper parts of wounds, and reduce the inflammatory response caused by infections and the damage caused by bacterial toxins to the wounds [26]. Combination therapy can have a synergistic effect, the use of photothermal, immunomodulation, and other modes of delivery combined with antibacterial, not only can control the infection, but also effectively improve the local environment of chronic diabetic wounds. Photothermal therapy can promote the healing of chronic wounds by promoting blood circulation, cell proliferation and migration, and bactericidal effect, but there is a risk of safety and thermal injury [27, 28]. In this study, we constructed a multifunctional photothermal platform, which can realize the effective treatment of chronic diabetic infected wounds and the removal of subcutaneous abscesses through the mild photothermal effect and immunomodulation, and showed excellent repair of chronic infected wounds. Effectiveness and potential [29].

**FIGURE 5:**
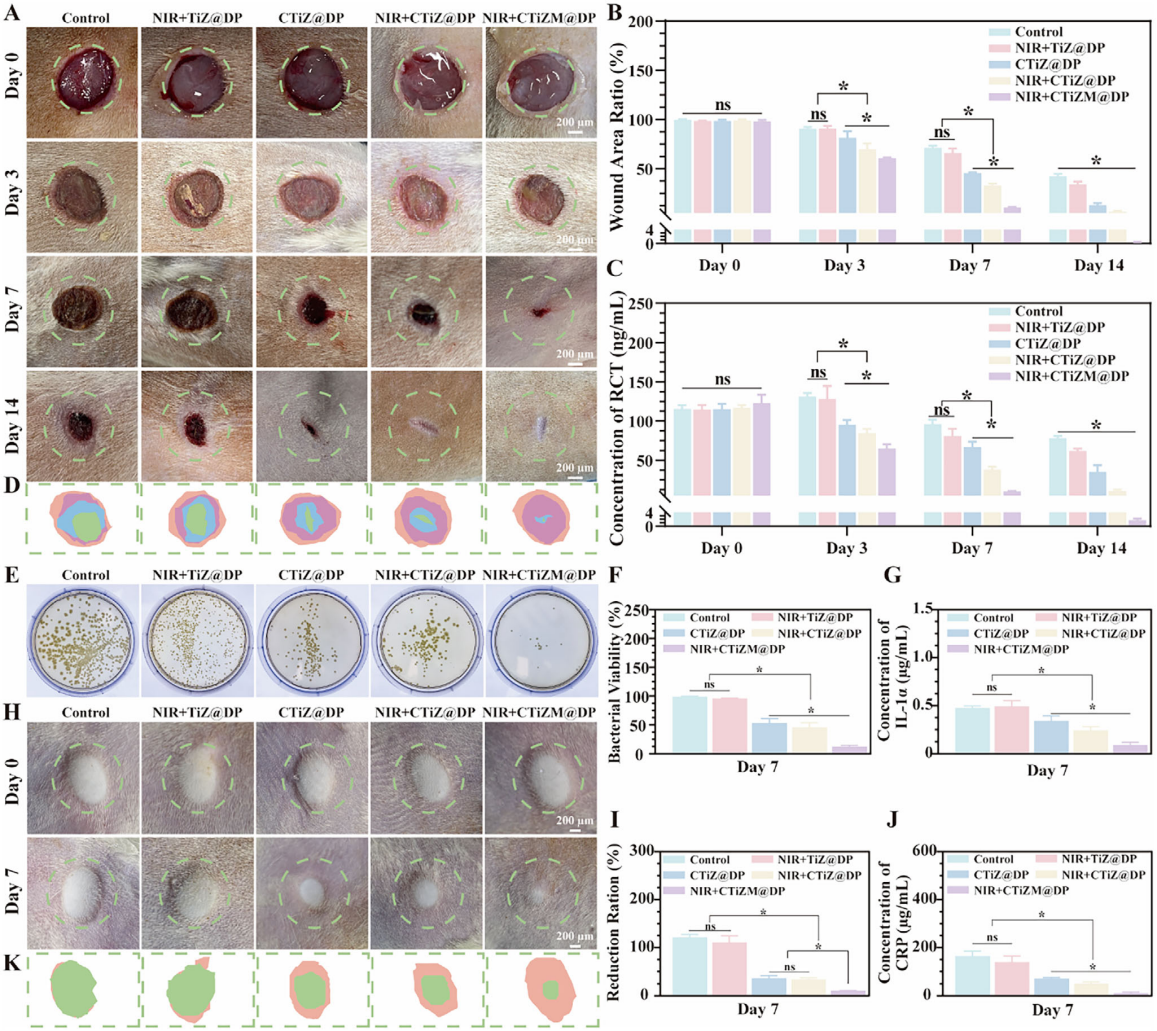
(A) Diabetic infected wound healing macrograph. (B) Statistical analysis of diabetic wound healing rate. (C) Calcitonin concentration in the blood of diabetic rats with diabetic wounds. (D) Schematic diagram of diabetic wound healing. (E) MRSA culture of wound secretions at 7 days of diabetic wounds. (F) Statistical analysis of MRSA viability; (G) IL‐1α concentration. (H) Appearance of diabetic rat subcutaneous abscess model. (I) Schematic diagram of the proportion of abscess reduction in the subcutaneous abscess model. (J)C‐reactive protein concentration in the blood of the diabetic rat subcutaneous abscess model. (Data represent independent experiments; all data are presented as mean ± SD with a sample size of n = 3; statistical analysis was performed using GraphPad Prism 9 software via one‐way analysis of variance (one‐way ANOVA); ns indicates not significant, *p < 0.05).

In this study, a chronic diabetic infected wound model and a chronic diabetic subcutaneous abscess model were constructed, respectively, and the antimicrobial‐promoting and repairing effects and MRSA clearance potential of CTiZM@DP hydrogel were tested. Firstly, a chronic diabetic infected wound model was established, and the healing of the wound was recorded at days 0, 3, 7, and 14 after surgery, and it was found that the wound healing was delayed at day 14 within the simple wound infection model group, while the wound as well as complete healing could be observed in the photothermal CTiZM@DP hydrogel group, and statistical analysis of the wound healing rate revealed that the photothermal CTiZM@DP hydrogel group The healing rate was significantly better than the other groups, and the wound healing was analyzed to find the process of continuous wound reduction (Figure 5A,B,D). The level of procalcitonin element (PCT) in the serum of rats was detected during the process of wound healing, which could indicate the status of infection within the rats, and it was found that the level of massive infection within the CTiZM@DP hydrogel group was significantly reduced compared with the other control groups, which demonstrated the effective antimicrobial effect of the CTiZM@DP hydrogel, which significantly reduced the level of infection (Figure 5C). In addition, at day 7, the secretion from the wound surface was taken for MRSA culture, and it was found that a large number of MRSA existed within the Control group, whereas there was basically no MRSA in the CTiZM@DP hydrogel group, and statistical analysis of the results revealed that the photothermal CTiZM@DP hydrogel had a significant antimicrobial effect (Figure 5E,F). To further evaluate the antimicrobial effect of CTiZM@DP hydrogel, a model of subcutaneous abscess was constructed under the skin of diabetic rats, and the size of subcutaneous abscess was recorded at day 0 and day 7 of the model respectively, and the size was analyzed, and the results could be observed that the abscesses were significantly reduced within the CTiZM@DP hydrogel group, and the statistically significant difference was found when compared with the other groups (Figure 5H,K,I). Further, to assess the effect of MRSA infection on the overall inflammatory state, IL‐1α and CRP in the blood were measured on day 7, and the results showed that CTiZM@DP hydrogel significantly reduced the level of inflammation in the blood, with statistically significant differences in the results when compared with the other groups (Figure 5G,J). In addition, liver and kidney function in rat blood was assessed 14 days post‐surgery, and the main organs were assessed. The results showed no differences in Alanine aminotransferase (ALT), aspartate aminotransferase (AST), creatinine (CRE), and blood urea nitrogen (BUN) levels compared to the normal group, as shown in Figure S15. Representative hematoxylin and eosin (H&E) staining images of the heart, liver, spleen, lung, and kidney from mice treated with different hydrogel formulations (Control, Hydrosorb, NIR+TiZ@DP, CTiZ@DP, NIR+CTiZ@DP, and NIR+CTiZM@DP) are shown in Figure S16. Across all treatment groups, no noticeable pathological abnormalities were observed in any major organ, indicating that the various hydrogel formulations did not induce observable systemic toxicity.

### 2.6 CTiZM@DP Photothermal Gel Promotes Epithelialization and Collagen Deposition for Wound Repair

The epithelial layer of normal skin is the first barrier of the organism against external pathogenic bacteria. The healing process of chronic infected wounds requires the removal of damaged tissues, then the continuous deposition of collagen to fill in the defective wound tissues, and finally, epithelialization is the final stage of wound repair to maintain the structure and function of the wound site. It has been found that transforming growth factor activates the Smad pathway in infected wounds to promote collagen synthesis and protease secretion, and promotes normal deposition and alignment of collagen in locally damaged tissues. Next, it promotes epithelial cell migration by stimulating fibroblasts to secrete a variety of epidermal growth factors and keratinocyte growth factors to cover the damaged tissue [30, 31].

**FIGURE 6:**
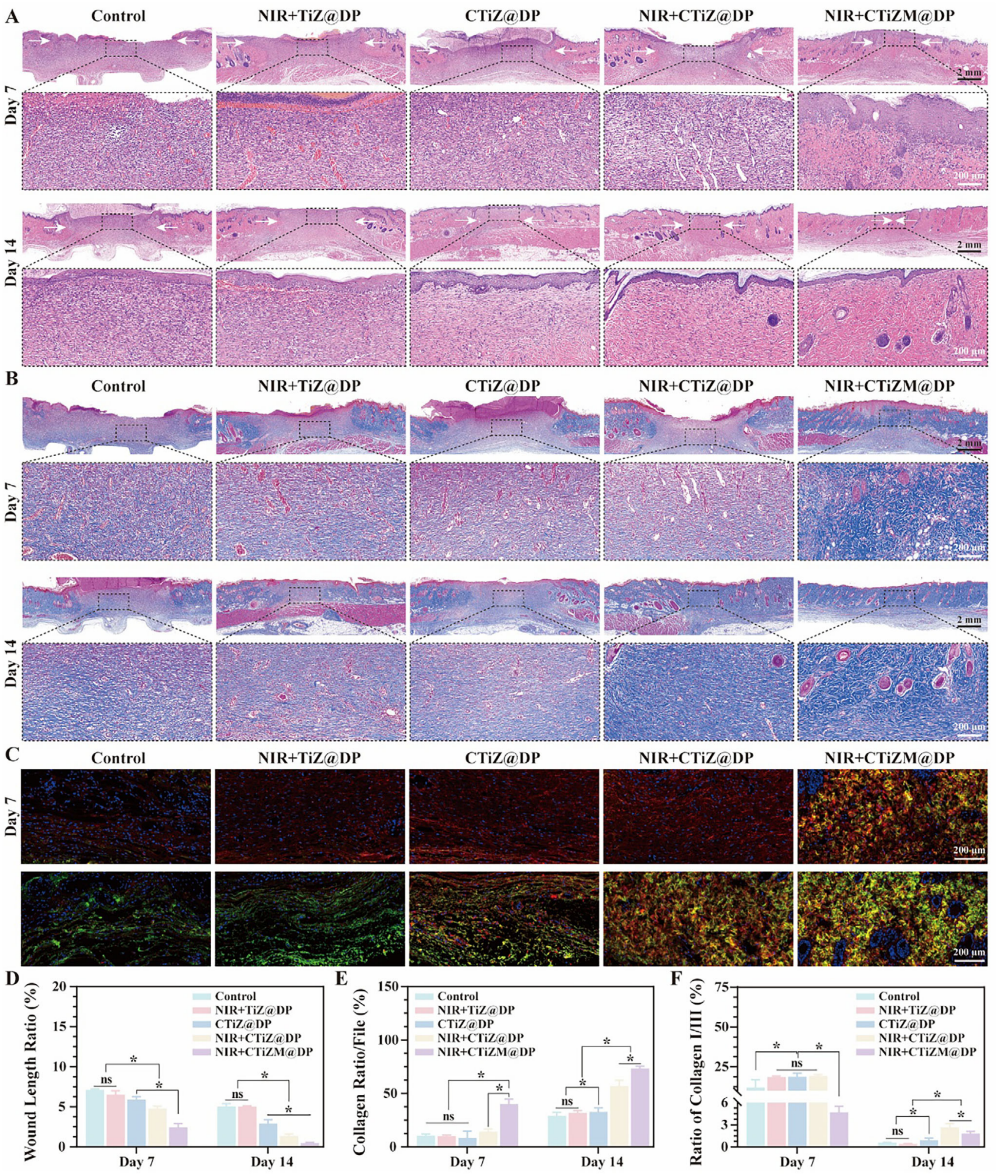
(A) H&E staining of diabetic wound tissue. (B) Masson staining of diabetic wound tissue. (C) Sirius red staining of diabetic wounds to label type I/III collagen. (D) H&E staining of wound length analysis. (E) Masson staining of collagen proportion statistical analysis. (F) Sirius red staining analysis of collagen type I/III proportion. (Data represent independent experiments; all data are presented as mean ± SD with a sample size of n = 3; statistical analysis was performed using GraphPad Prism 9 software via one‐way analysis of variance (one‐way ANOVA); ns indicates not significant, ^*^ *p* < 0.05).

In this study, the epithelialization of wound healing was first assessed, and wound closure was examined using H&E staining, which was performed at 7 and 14 days after treatment, respectively, and it was found that the length of wounds in the CTiZM@DP light and hot water gel group was significantly lower than that of the other control groups, and wound healing was increased with the increase of time, and in the high magnification images of the tissue sections, the CTiZM@DP light and hot water More hair follicle tissues were observed in the CTiZM@DP Light Hot Water Gel group (Figure 6A,D), indicating that the CTiZM@DP Light Hot Water Gel group had better epithelialization and more skin appendages. Next, the collagen deposition during wound healing was evaluated using Masson staining, and it was found that more collagen was deposited in the wound site by CTiZM@DP Photothermal Gel at day 7, and with the increase of time, more of its collagen was deposited by day 14, and the arrangement of the collagen was more close to the structure of the physiological skin, and the proportion of collagen was statistically analyzed, and the results were found to be comparable to those of other groups The results were statistically analyzed for the proportion of collagen and found that the results were significantly different from the other groups (Figure 6B,E). Finally, the distribution and proportion of collagen were further evaluated by Sirius red staining, and the results showed that there was a red type I collagen and a green type III collagen in the healing process of wounds, and that there was a lower proportion of type I collagen in the photothermal CTiZM@DP photo‐hydrothermal gel group at 7 days, and that there was a greater proportion of type I collagen in the other groups. Collagen, with the increase of time, the proportion of type III collagen gradually increased, at 14 days in the photothermal CTiZM@DP hydrothermal gel group within the type I/III collagen more close to the normal skin structure, the proportion of statistical analysis found that the CTiZM@DP photothermal water gel group compared with the other groups of the results of the results of the significant difference (Figure 6C,F).

### 2.7 CTiZM@DP Photo‐Hydrothermal Hydrogel Promotes Wound Healing by Modulating the Immune Microenvironment Through Inhibition of the NF‐κB Pathway

In diabetic chronic infected wounds, MRSA infection and hyperglycemic environment can lead to tissue cell damage, and the activated immune response can continuously upregulate NF‐κB expression, resulting in difficult‐to‐control inflammation and prolonged wound healing [32]. Meanwhile, the continuous upregulation of NF‐κB can also affect the function of fibroblasts, inhibit their proliferation and migration, change the expression and distribution of fibroblasts, and reduce the synthesis of ECM. In addition, NF‐κB also affects the proliferation and migration of vascular endothelial cells, damages vascular endothelial cells through the activation of excessive inflammatory factors, and inhibits blood vessel formation. It was found that inhibition of excessive NF‐κB expression during the healing process of diabetic chronic infected wounds could promote the wound repair process [33, 34].

**FIGURE 7:**
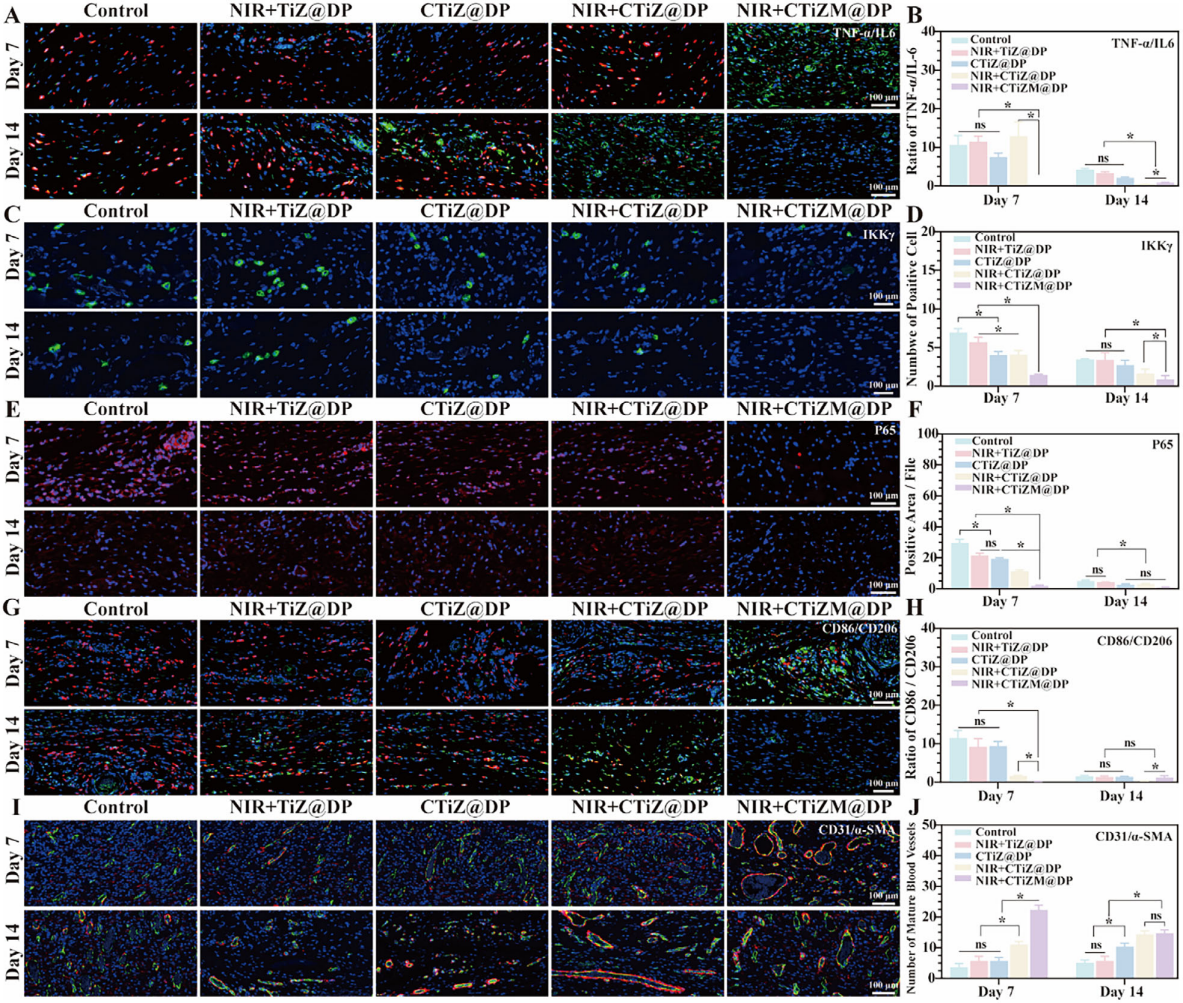
(A) Expression levels of tumor necrosis factor‐α (TNF‐α) and interleukin‐6 (IL‐6) in diabetic wound tissues; IL‐6 is labeled with green fluorescence, TNF‐α with red fluorescence, and nuclei with blue fluorescence. (B) Quantitative analysis evaluating the ratio of TNF‐α–positive area to IL‐6–positive area. (C) Expression level of IκB kinase γ (IKKγ) in diabetic wound tissues; IKKγ is labeled with green fluorescence, and nuclei with blue fluorescence. (D) Quantitative analysis assessing the number of IKKγ‐positive cells. (E) Expression level of P16INK4a in diabetic wound tissues; P16INK4a is labeled with red fluorescence, and nuclei with blue fluorescence. (F) Quantitative analysis evaluating the proportion of P65‐positive area. (G) Expression levels of CD86 and CD206 in diabetic wound tissues; CD206 is labeled with green fluorescence, CD86 with red fluorescence, and nuclei with blue fluorescence. (H) Quantitative analysis of the ratio of CD86‐positive area to CD206‐positive area. (I) Expression levels of CD31 and α‐smooth muscle actin (α‐SMA) in diabetic wound tissues; CD31 is labeled with green fluorescence, α‐SMA with red fluorescence, and nuclei with blue fluorescence. (J) Quantitative analysis of the number of mature blood vessels. (Data represent independent experiments; all data are presented as mean ± SD with a sample size of n = 3; statistical analysis was performed using GraphPad Prism 9 software via one‐way analysis of variance (one‐way ANOVA); ns indicates not significant, ^*^ *p* < 0.05).

Therefore, we first examined the expression levels of the inflammatory cytokines TNF‐α and IL‐6 on days 7 and 14. The results showed that on day 7, the red fluorescence–labeled TNF‐α was markedly reduced in the photothermal CTiZM@DP group, whereas a strong green fluorescence signal corresponding to IL‐6 was observed, indicating that the photothermal CTiZM@DP group had already progressed into the repair stage. By day 14, only minimal IL‐6 expression was detected in this group, while large amounts of TNF‐α and IL‐6 were still present in the other groups, demonstrating that the wounds treated with the photothermal CTiZM@DP hydrogel had essentially healed. The ratio of TNF‐α to IL‐6 showed significant statistical differences among groups (Figure 7A,B). To further investigate the mechanisms underlying the altered wound‐healing microenvironment, we assessed the expression of IKKγ, a key regulator of the NF‐κB signaling pathway. The results revealed a time‐dependent decrease in IKKγ expression, with the photothermal CTiZM@DP hydrogel group exhibiting the lowest levels on both days 7 and 14. Quantification of IKKγ‐positive cells confirmed significant intergroup differences (Figure 7C,D). Diabetic chronic infected wounds commonly display features of cellular senescence, which may be associated with hyperglycemia‐induced and inflammation‐driven upregulation of NF‐κB. Previous studies have demonstrated that NF‐κB can directly or indirectly regulate the expression of the senescence marker P16INK4a, leading to cell‐cycle arrest of immune and reparative cells at the wound site, thereby exacerbating chronic disease progression. Inhibition of NF‐κB has been reported to alleviate the development of atherosclerosis and neurodegenerative diseases. Consistently, our study also identified abundant inflammatory senescence phenotypes in diabetic MRSA‐infected chronic wounds (Figure S17).

Then, We therefore evaluated the senescence status of the wound tissue on days 7 and 14. The results indicated that the photothermal CTiZM@DP hydrogel significantly reduced the expression of the senescence‐associated marker P65 at the wound site, with levels markedly lower than those in the control groups and further decreasing over time. Statistical analysis of the positive area confirmed significant intergroup differences. These findings suggest that the photothermal CTiZM@DP hydrogel effectively mitigates hyperglycemia‐ and MRSA‐induced senescence phenotypes and promotes wound healing (Figure 7E,F). Clearance of senescent cells can remodel the immune microenvironment and facilitate wound repair. Thus, we further assessed post‐healing immune microenvironmental changes by identifying macrophage phenotypes. On day 7, the photothermal CTiZM@DP hydrogel group predominantly exhibited an M2 macrophage phenotype, characterized by strong green fluorescence of CD206 and minimal red fluorescence of CD86, whereas the other groups mainly displayed an M1 phenotype. This pattern indicated that the photothermal hydrogel group had transitioned into the reparative phase. With time, the other groups gradually shifted toward an M2‐dominant phenotype, whereas macrophage activity in the photothermal CTiZM@DP hydrogel group became minimal, suggesting completion of tissue repair. Statistical analysis confirmed significant differences among groups (Figure 7G,H). Another crucial factor contributing to the impaired healing of diabetic MRSA‐infected chronic wounds is endothelial cell damage caused by hyperglycemia, excessive inflammation, and senescence, leading to reduced angiogenesis. Therefore, angiogenic capacity was further evaluated in this study. On days 7 and 14, abundant mature blood vessels double‐positive for CD31 and α‐SMA were observed in the photothermal CTiZM@DP hydrogel group, significantly exceeding those in the control groups. Quantitative analysis also revealed significant differences among groups, demonstrating that the photothermal CTiZM@DP hydrogel improved the local repair microenvironment and provided favorable blood‐supply conditions to support wound healing (Figure 7I,J).

## 3 Conclusion

Given the current limitations of traditional therapies for chronic MRSA‐infected wounds, it is crucial to develop effective treatment methods for such wounds. The approach proposed in this study, which involves synthesizing TiZ nanoparticles, transforming them into CTiZM‐targeted nanoparticles, and encapsulating them in a temperature‐sensitive hydrogel to form CTiZM@DP, provides a promising solution. This strategy not only overcomes the disadvantages of traditional antibiotic treatments, such as lack of targeting and potential systemic toxicity, but also takes advantage of the unique properties of nanomaterials and cell‐based targeting, realizing a treatment strategy that integrates mild photothermal effect, precise targeting, antibacterial, and immunomodulatory functions. The CTiZM@DP composite gel has demonstrated significant efficacy in targeted sterilization both inside and outside macrophages in chronic diabetic wounds of rats, thereby alleviating pathological damage and inflammation. Moreover, it is beneficial to the restoration of macrophage function, the remodeling of the immune and regenerative microenvironments, and ultimately promotes wound healing. This novel therapeutic tool has the potential to significantly improve the management of drug‐sensitive and drug‐resistant bacteria in the clinical environment, providing a new direction for the treatment of chronic diabetic wounds.

## 4 Materials and Methods

### 4.1 Materials

Mouse Peripheral Blood Neutrophil Cells were obtained from Wuhan Procell System Technology Co., Ltd., RAW 264.7 (RRID: CVCL_0493), and 3T3‐L1 cell lines (RRID: CVCL_0123, Shanghai Cell Bank, Chinese Academy of Sciences, China), and Methicillin‐Resistant Staphylococcus aureus were used as experimental models. Culture media included high‐glucose DMEM (containing 10% fetal bovine serum, Gibco, USA) and nutrient broth and LB medium (Thermo Fisher Scientific, USA). Key reagents included Ti_3_AlC_2_ MAX powder (Sinopharm Chemical Reagent Co., Ltd, China), lithium fluoride, sodium hydroxide, paraformaldehyde, hydrofluoric acid, anhydrous ethanol, and chlorogenic acid (Sigma‐Aldrich, USA; Aladdin, China).

### 4.2 Bacterial Strains and Cell Lines

In this research, Methicillin‐Resistant Staphylococcus aureus (Bena Culture Collection, BNCC330041, Beijing, China) is selected. Regarding cell lines, the mouse mononuclear macrophage leukemia cell line RAW264.7 and the mouse embryonic fibroblast cell line 3T3‐L1 are employed. All experiments related to MRSA are carried out in laboratories that meet biosafety conditions. Cell lines are all purchased from the Shanghai Cell Bank of the Chinese Academy of Sciences (China). Bacteria are cultured and amplified using nutrient broth medium and LB medium (Luria‐Bertani medium, Thermo Fisher Scientific, USA). Cell lines are all cultured in high‐glucose DMEM medium containing 10% fetal bovine serum (FBS, Gibco, USA), 1% penicillin and streptomycin (Thermo Fisher Scientific, USA), and are cultured in an incubator with 5% CO_2_ and 95% air under sterile conditions.

### 4.3 Fabrication and Characterization of TiZM Nanospheres

#### 4.3.1 Preparation of Single‐Layer Ti_3_C_2_ Nanosheets

First, prepare raw materials such as Ti_3_AlC_2_ MAX powder, lithium fluoride, hydrochloric acid, and deionized water. Next, dissolve 2.0 g of LiF in 40 mL of 9M HCl solution and stir at room temperature for 30 min. Slowly add 2.0 g of Ti_3_AlC_2_ MAX powder and then stir at 35°C with a speed of 400 rpm for 24 h. Wash and centrifuge with deionized water until the pH of the supernatant is approximately 6. Re‐disperse the precipitate with ethanol and conduct ultrasonic intercalation in an ice‐water bath for 1 h. Then, obtain several layers of Ti_3_AlC_2_T_x_ MAX flakes through centrifugation and freeze‐drying, and prepare a 2mg/mL Ti_3_AlC_2_T_x_ MAX colloidal dispersion. Subsequently, sonicate the dispersion for 1 h and let it stand at room temperature for 24 h to make the flakes precipitate. Pour out the supernatant and wash the precipitate with deionized water several times. Finally, dry the precipitate in a vacuum to obtain the single‐layer Ti_3_C_2_ nanosheets [35].

#### 4.3.2 Preparation of TiZ Nanoparticles

Disperse 100 mg of SiO_2_ nanospheres in 10 mL of Ti_3_C_2_ MXene dispersion and sonicate for 10 min, followed by stirring for 1 h to ensure uniform coating of Ti_3_C_2_ on the SiO_2_ surface. Centrifuge, wash with deionized water, and dry to obtain the SiO_2_@Ti_3_C_2_ core‐shell structure. Dissolve 1.0 g of ZnAl‐LDH nanosheets in 100 mL of deionized water, then disperse the SiO_2_@Ti_3_C_2_ core‐shell structure in this solution and adjust the pH to approximately 10 using NaOH. Stir at 80°C for 12 h to complete the reaction. After reaction, centrifuge and wash with deionized water. Disperse the precipitate in a 5 wt.% HF solution, react for 6 h to dissolve the SiO_2_ template, generating a hollow‐structured TiZ nanoparticle. Wash multiple times with deionized water and dry under vacuum for storage [35, 36].

#### 4.3.3 Preparation of CTiZM Nanoparticles

Dissolve 50 mg of chlorogenic acid in 10 mL of deionized water. Disperse 100 mg of TiZ nanoparticles into the chlorogenic acid solution and sonicate for 10 min. Stir the mixture at room temperature for 24 h, then centrifuge and wash with deionized water. Neutrophils were stimulated with PSM‐β, and the apoptosis level after intervention was identified by flow cytometry. After incubation, the cells were washed with PBS to remove any residual PSM‐β. The cells were lysed with 0.25% trypsin and centrifuged at 12 000g for 10 min at 4°C to remove cell nuclei and large organelles. The supernatant was collected and centrifuged at 20 000g for 30 min to remove cytoplasmic components. The precipitated membrane fractions were collected and further purified by ultracentrifugation at 100 000g for 1 h to obtain highly pure neutrophil membranes from early apoptotic cells. Neutrophil membranes were mixed with CTiZ nanoparticles at a mass ratio of 1:1 in PBS. The mixture was extruded through a membrane with 100–200 nm pores for 10–15 times to ensure uniform coating. After extrusion, the product was centrifuged to remove uncoated membrane fragments; the final product was washed and dispersed in PBS to obtain neutrophil membrane‐coated CTiZM nanoparticles [37].

#### 4.3.4 SEM and TEM

Disperse 10 mg of TiZ and CTiZM nanoparticles, respectively, in anhydrous ethanol and sonicate for 10 min to ensure uniform dispersion of the nanoparticles. Drop a small amount of the dispersion onto a copper substrate and dry it in a 60°C oven. After sputter‐coating with gold, observe the surface morphology under the SEM (JEOL JSM‐7500F, Japan) and TEM (JEOL JEM‐2010, Japan), and then perform elemental analysis under the TEM [35, 37].

#### 4.3.5 UV–vis Absorption Spectroscopy

Place Ti_3_C_2_ and TiZ nanomaterials separately in the sample cell of the UV‐Vis (Lambda 950, PerkinElmer, USA) absorption spectrometer, perform scanning, and observe changes in absorbance [38].

#### 4.3.6 FTIR

According to the steps in the literature, take 10 mg of powder to prepare the sample and place it on the FTIR spectrometer sample holder. Set the scan range from 4000 to 400 cm^−^^1^, and record the FTIR (Nicolet 6700, Thermo Fisher Scientific, USA) spectrum of TiZ nanoparticles to analyze the surface functional group characteristics [36].

#### 4.3.7 XPS

According to the steps in the literature, take 10 mg of powder to prepare the sample, ensuring sufficient surface exposure. Place the sample on the XPS (Escalab 250Xi, Thermo Fisher Scientific, USA) sample stage and evacuate to ultra‐high vacuum (UHV) conditions. Set the parameters to identify all elements in the sample, perform a high‐resolution scan of the Zn, Al, and Ti elements, and analyze their characteristic binding energy region [36].

#### 4.3.8 Detection of Specific Membrane Proteins on Neutrophils' Surface

Follow the procedure outlined in the referenced literature. First, fix bacteria‐infected Neutrophils with 4% paraformaldehyde (Sigma‐Aldrich, USA) for 1 h. After washing with PBS (Thermo Fisher Scientific, USA), block the cells overnight with an immunofluorescence blocking solution. Use a primary antibody against TLR4 (Abcam, UK) and an FITC‐labeled secondary antibody (Abcam, UK) to label TLR4, and a primary antibody against Siglec F (Abcam, UK) with a CY3‐labeled secondary antibody (Abcam, UK) to label Siglec F. Stain the cells with DAPI. Evaluate fluorescence expression using a fluorescence microscope and flow cytometry [39].

#### 4.3.9 Bacterial Targeting

Infect macrophages with MRSA for 24 h, then add different Rhodamine‐labeled CTiZM nanoparticles and co‐incubate for 48 h. Stain the bacteria according to the FITC‐D‐Lys instructions (Guangzhou Wehua Biotechnology Co., Ltd., China), then fix with 4% paraformaldehyde. Stain the nuclei with DAPI and perform colocalization analysis of nanoparticles and bacteria under a laser confocal microscope (Leica, Germany).

### 4.4 Fabrication and Characterization of CTiZM@DP Hydrogel

#### 4.4.1 Fabrication of DOPA‐PNIPAm Hydrogel

The hydrogel synthesis procedure is based on the steps provided in the reference [40].

#### 4.4.2 In Vitro Photothermal Imaging

Place 200 µg of CTiM (400 µg /ml, 500 µl) on the bottom of a 24‐well plate. After spreading it evenly, irradiate it with a near‐infrared light emitter at 808 nm. Set the power at 1.5 W/cm^2^ and irradiate for 180 s. Moreover, use a photothermal imager to conduct photothermal imaging of the temperature on the bottom of the well plate. Record the photothermal images of different groups at 15, 30, 60, 90, 120, and 180 s, respectively.

#### 4.4.3 SEM

Prepare DP photothermal hydrogels containing different nanoparticles. Weigh 10 mg of the composite hydrogel and place it in a sterile cell culture dish. Then, place it in a freeze‐dryer for vacuum freeze‐drying for 48 h. Subsequently, use liquid nitrogen embrittlement to expose the cross‐sections of different hydrogels. Spray gold on the surface materials and observe under the SEM microscope according to the methods described in the literature. Collect the ultrastructure images and simultaneously conduct the collection and analysis of the elemental distribution situation.

#### 4.4.4 Evaluation of Hydrogel Phase Transition and Infrared Light Penetration Performance

Take 1 mL of CTiZM@DP composite hydrogel and place it in an EP tube. Irradiate the EP tube with an 808‐nm laser until the temperatures reach 25°C, 37°C, and 45°C, respectively, and use a digital camera to photograph the gel‐forming condition of the hydrogel. Next, place the EP tube on the desktop with a white backboard as the background. Continuously irradiate it with an 808‐nm laser. When heated to 25°C, 37°C, and 45°C, use a digital camera to record the intensity of the laser penetrating the white backboard.

#### 4.4.5 Evaluation of the Photothermal Capacity of CTiZM@DP Composite Hydrogel

According to the above experimental procedures, record the temperature images of CTiZM@DP hydrogel under 808‐nm laser irradiation at different time points. Moreover, irradiate with the laser for 600 s and record the temperature change every 30 s. Change the power of laser irradiation to 0.5, 1.0, 1.5, and 2.0 W/cm^2^, respectively, and record the temperature change at different time points.

#### 4.4.6 Bacterial Viability and Dead Bacteria Staining

Use the SYTO 9/PI staining kit (Thermo Fisher Scientific, USA) to detect the viability and death of MRSA according to the instruction manual. Co‐culture 1 mL of MRSA liquid with CTiZM@DP hydrogel for 12 h, and then irradiate with an 808‐nm laser for 120 s. Subsequently, add SYTO 9/PI staining solution to the mixed solution. After incubation for 30 min, observe using confocal laser scanning microscopy.

#### 4.4.7 MRSA TEM Detection

Co‐culture 1 mL of MRSA suspension with CTiZM@DP hydrogel for 12 h, and then irradiate with an 808‐nm laser for 120 s. Subsequently, add SYTO 9/PI staining solution to the mixed solution. After incubation for 30 min, centrifuge at 300 g for 10 min. Take the precipitate and add 2.5% glutaraldehyde (Sigma‐Aldrich, USA). Let it stand at room temperature for 1 h and then at 4°C for 24 h. Observe the ultrastructure of the MRSA by TEM according to the methods described in the above‐mentioned references.

### 4.5 Evaluation of Senescent Macrophage Phenotypes and Immune Remodeling

#### 4.5.1 Mitochondrial Membrane Potential

Evaluate the mitochondrial membrane potential of macrophages according to the instruction manual. First, induce macrophage senescence by culturing with MRSA for 24 h. Then, co‐culture the nanoparticles and hydrogels of different groups with senescent macrophages for 48 h. Subsequently, use the JC‐1 probe (Beyotime, China) to detect the mitochondrial membrane potential. Dilute JC‐1 with sterile ddH_2_O, and then add it to the macrophages and incubate for half an h. Observe the red and green fluorescence in the macrophages under a confocal microscope (Leica Microsystems, Germany).

#### 4.5.2 ROS Detection

Culture senescent macrophages in the manner described in the above mitochondrial membrane potential section. Then, use DCFH‐DA reactive oxygen species probe (Beyotime, China) to detect the level of reactive oxygen species in macrophages. After incubating the cells with DCFH‐DA for half an h, add Hoechst stain for 5 min to label the cell nuclei. Collect bright‐field and fluorescence images under a fluorescence microscope.

#### 4.5.3 β‐Galactosidase Staining

Culture macrophages according to the above‐mentioned steps to evaluate the intracellular senescence level of macrophages. Prepare the β‐galactosidase staining (Sigma‐Aldrich, USA) substrate according to the Sigma instructions. Add it into the cells, avoid light and CO_2_, and incubate at 37°C overnight. Then, observe the β‐galactosidase staining of senescent cells under a fluorescence microscope.

#### 4.5.4 Immunofluorescence

Detect the expression levels of IL‐4 and IL‐1α in macrophages after MRSA infection, respectively. Culture macrophages after bacterial infection according to the steps described above. Then, fix them with 4% paraformaldehyde for 30 min. Wash them with PBS three times, and then block them with an immunological blocking solution for 24 h. After washing with PBS three times, add primary antibodies of rabbit‐sourced IL‐4 (Cell Signaling Technology, USA) and IL‐1α (Cell Signaling Technology, USA), respectively. After incubation for 24 h, wash with PBS three times, and then add goat anti‐rabbit secondary antibodies labeled with RTIBC (Sigma‐Aldrich, USA), respectively. After incubation for 24 h, wash with PBS three times. Then add FITC‐labeled F‐actin (Sigma‐Aldrich, USA) and DAPI (Sigma‐Aldrich, USA), respectively, and incubate for 1 h and 5 min, respectively. After washing with PBS, use confocal laser scanning microscopy for image acquisition and observation. Next, identify the macrophage phenotypes using the same steps. Replace the primary antibodies with CD86 (Abcam, UK) and CD206 (Abcam, UK), respectively, and use FITC‐labeled rabbit‐sourced secondary antibodies (Sigma‐Aldrich, USA) to label CD86 and RBITC‐labeled rabbit‐sourced secondary antibodies (Sigma‐Aldrich, USA) to label CD206, respectively.

### 4.6 Phenotypic Regulation and Microenvironment Regulation of Fibroblasts

#### 4.6.1 ROS Detection

Construct a co‐culture system of macrophages and fibroblasts after MRSA infection. Prepare macrophages after bacterial infection according to the above‐mentioned steps. Establish a co‐culture system using Transwell. In the upper chamber, culture macrophages at a density of 1 × 10^4^ per well, and in the lower chamber, culture fibroblasts at a density of 1 × 10^5^ per well. Add different types of nanoparticles and hydrogels to the upper chamber. After 48‐h co‐culture, take the fibroblasts in the lower chamber for ROS level detection. Conduct staining and image acquisition according to the steps described in 4.4 above.

#### 4.6.2 Immunofluorescence

Evaluate the expression levels of TNF‐α, α‐SMA, and Collagen I/Collagen III in fibroblasts, respectively. First, collect fibroblasts according to the steps described above. Use rabbit‐sourced TNF‐α primary antibody (Abcam, UK) and RBITC‐labeled goat anti‐rabbit secondary antibody (Sigma‐Aldrich, USA) to label TNF‐α, and use DAPI to label the cell nucleus. Next, use rabbit‐sourced α‐SMA primary antibody (Abcam, UK) and FITC‐labeled goat anti‐rabbit secondary antibody (Sigma‐Aldrich, USA) to label α‐SMA, respectively, and use DAPI (Sigma‐Aldrich, USA) to label the cell nucleus. Finally, use Collagen I primary antibody (Abcam, UK) and RBITC‐labeled secondary antibody (Sigma‐Aldrich, USA) to label Collagen I, and use Collagen III primary antibody (Abcam, UK) and FITC‐labeled secondary antibody (Sigma‐Aldrich, USA) to label Collagen III respectively, with DAPI (Sigma‐Aldrich, USA) used to label the cell nucleus. Use confocal laser scanning microscopy to acquire images and evaluate the expression of different proteins.

### 4.7 Evaluation of Promoting Wound Healing and Antibacterial Ability in Vivo

#### 4.7.1 Animal Model

In this study, Sprague‐Dawley rats were selected and purchased from and housed in the Animal Experiment Center of Sichuan University. All experimental operations conformed to animal ethics and were approved by the Animal Care and Experimental Committee of West China Hospital, Sichuan University (ethical approval number: 20240221041). First, SD rats weighing 3000 g were selected and adaptively housed in an SPF environment for 2 weeks. Next, the diabetic rat model was induced by intraperitoneal injection of streptozotocin (Sigma‐Aldrich, USA) at a dose of 50 mg/kg body weight. After 72 h, the blood glucose of the rats was monitored. If the blood glucose was continuously higher than 16.7 mmol/L and symptoms such as polydipsia, polyphagia, polyuria, and weight loss appeared, the model was judged to be successfully established. Next, an infectious wound model was constructed. First, the rats were anesthetized with isoflurane, fixed on the operating table for skin preparation, and disinfection of the back. Then, a 1‐cm‐diameter circular skin defect was made using a skin punch. Staphylococcus aureus at a concentration of 1 × 10^7^ CFU/mL each were selected, and 0.1 mL of the mixed MRSA suspension was evenly smeared on the wound. After 24 h, the wound bacterial fluid was smeared on a plate to determine the success of the model. Then, the following groups were set up: 1. Diabetic infectious wound group; 2. Photothermal TiZ@DP hydrogel group; 3. CTiZ@DP hydrogel group; 4. Photothermal CTiZ@DP hydrogel group; 5. Photothermal CTiZM@DP hydrogel group. Different groups of hydrogels were coated on the surface of the diabetic infectious wound, respectively. After the operation, the rats were individually housed in an SPF environment, and diabetic rat‐specific feed and drinking water were provided. Postoperatively, wounds in the hydrogel + NIR group were covered with the hydrogel and subsequently exposed to NIR irradiation for 10 min per day over a period of five consecutive days. The wound healing of the rats was regularly observed. Wound tissue fluid, blood, and tissues were taken at 7 and 14 days, respectively, for wound healing evaluation.

#### 4.7.2 RCT Detection

Use the enzyme‐linked immunosorbent assay (ELISA) kit of MyBioSource company to detect Rat Calcitonin (RCT, MyBioSource, USA) in rat blood. First, collect 0.5–1 mL of blood from the tail vein into a centrifuge tube containing an anticoagulant. Centrifuge at 3000–4000 rpm at 4°C for 10–15 min to obtain plasma. Prepare standard substances, enzyme‐labeled antibodies, substrate solutions, stop solutions, etc., according to the kit instructions. Add the standard substances and samples into the antibody‐coated plate. Wash after incubation at 37°C for 1–2 h. Then add the enzyme‐labeled antibodies. Wash again after incubation at 37°C for 1–2 h. Add the substrate solution. Incubate at 37°C in the dark for 15‐30 min for color development. Add the stop solution to terminate the reaction. Measure the absorbance at 450 nm (BioTek, USA) using a microplate reader. Check the RCT concentration of the sample according to the standard curve.

#### 4.7.3 Skin Subcutaneous Abscess Model

Select healthy SD rats weighing 300 g and acclimate them for 2 weeks. Anesthetize the rats with isoflurane and then fix them on the operating table. Shave the hair at the predetermined modeling site and disinfect with iodophor. Select MRSA, culture it until the logarithmic growth phase, centrifuge and wash it, and then prepare a suspension with a concentration of 1 × 10^8^ CFU/mL using sterile normal saline. Use a sterile syringe to draw 0.5 mL of the suspension and inject it subcutaneously at the disinfected skin site. 24 h after the operation, inject 0.5 mL of different hydrogels according to the above‐mentioned animal experimental groups. Housing the animals separately and observing the skin changes at the injection site after 7 days.

#### 4.7.4 Evaluation of IL‐1α and CRP Levels

7 days after constructing a subcutaneous abscess model in rats, collect 2 mL of blood from the tail vein into a centrifuge tube containing an anticoagulant. For the plasma sample, centrifuge at 4000 rpm at 4°C for 15 min to obtain it. For the serum sample, allow the blood to stand at room temperature for 1–2 h and then centrifuge under the same conditions. Dilute the standard substances according to the steps of the ELISA kit of the R&D company, and then add samples, incubate, wash, add enzyme‐labeled antibodies, develop color, terminate the reaction, and finally use a microplate reader to detect the concentration levels of IL‐1α and CRP.

### 4.8 Evaluation of Epithelialization and Collagen Formation on the Wound Surface

#### 4.8.1 H&E Staining

According to the steps described in the literature [41], use the staining kit from Servicebio (Wuhan Servicebio Technology Co., Ltd, China). Tissue samples from the wound surface were collected on the 7th and 14th days after the operation, respectively. The fresh wound‐surface tissues were placed in sufficient 10% neutral formalin for 24‐h fixation. Subsequently, the tissues were dehydrated successively in 70%, 80%, 90%, 95% and 100% ethanol (twice) for 2 h each, and then placed in xylene for 2‐h clearing. Then, the tissues were infiltrated with wax in a constant‐temperature box at around 60°C to form tissue paraffin blocks. The paraffin blocks were cut into 5‐µm‐thick slices using a microtome. The slices were then floated on the surface of warm water at 50°C to be flattened and picked up with glass slides and mounted onto the slides. After that, the slides were placed in xylene for 15‐min dewaxing (with the xylene changed twice). Then, the slides were rehydrated successively in 100%, 95%, 90%, 80% and 70% ethanol for 2 min each. The rehydrated slides were stained in hematoxylin staining solution for 15 min. The stained slides were differentiated in 1% hydrochloric acid‐ethanol differentiating solution for 10 s, and then placed in saturated lithium carbonate solution for 2 min, blueing‐back. Next, the slides were stained in 0.5% eosin staining solution for 5 min. Finally, after dehydration (successively in 100%, 95%, 90%, 80% and 70% ethanol for 2 min each) and clearing in xylene for 2 min, the slides were coverslipped and image collection was performed under a microscope (Olympus, Japan).

#### 4.8.2 Masson Staining

Prepare paraffin sections and hydrate them according to the steps described in H&E, use the staining kit from Servicebio (Wuhan Servicebio Technology Co., Ltd, China). Then, stain with hematoxylin for 10 min, followed by rinsing with running water. Differentiate with 1% hydrochloric acid‐ethanol for several seconds and then rinse with running water. Soak the sections in saturated lithium carbonate aqueous solution for 1–2 min for bleaching back and then rinse. Place the sections in ponceau‐acid fuchsin solution and stain for 10 min, followed by a brief rinse. Then, place the sections in phosphomolybdic acid solution for 5 min and then rinse. Put the sections in aniline blue solution and stain for 5 min, followed by a quick rinse. Finally, dehydrate, clear, and mount the sections. After drying, images can be collected under a microscope [42].

#### 4.8.3 Sirius Red Staining

First, prepare the sections according to the dewaxing to water steps of H&E staining, using the staining kit from Servicebio (Wuhan Servicebio Technology Co., Ltd, China). Then, place the sections in a 0.1% Sirius red saturated picric acid solution for 1–2 h of staining, followed by rapid rinsing with distilled water. Subsequently, dehydrate the sections successively with 95% and 100% ethanol, and clear them with xylene I and xylene II. Finally, mount the sections with neutral balsam. After drying, images can be collected under a polarizing microscope to observe the collagen fibers. Different types of collagen fibers show different colors under polarized light [42].

### 4.9 NF‐κB Mediated Immune Remodeling

#### 4.9.1 Immunofluorescence Staining

First, prepare the sections according to the dewaxing to water steps of H&E staining. Then, perform antigen retrieval by microwave heating and cooling in citrate buffer. Next, block with PBS buffer containing normal serum and Triton X‐100 at room temperature for 1 h, and then incubate with the primary antibody. In this study, key indicators of NF‐κB‐mediated immunoregulation were evaluated. First, TN‐α primary antibody (Abcam, UK) and CY3‐labeled secondary antibody were used to label TNF‐α, and IL‐6 primary antibody(Abcam, UK) and FITC‐labeled secondary antibody (Sigma‐Aldrich, USA) were used to label IL‐6. Next, IKKγ primary antibody and FITC‐labeled secondary antibody (Sigma‐Aldrich, USA) were used to label IKKγ. Then, P65 primary antibody (Abcam, UK) and CY3‐labeled secondary antibody were used to label P65. Then, CD86 primary antibody (Abcam, UK) and CY3‐labeled secondary antibody (Sigma‐Aldrich, USA) were used to label CD86, and CD206 primary antibody (Abcam, UK) and FITC‐labeled secondary antibody were used to label CD206. Finally, stain the vascular‐related indicators. α‐SMA primary antibody (Abcam, UK) and CY3‐labeled secondary antibody (Sigma‐Aldrich, USA) were used to label α‐SMA, and CD31 primary antibody and FITC‐labeled secondary antibody (Sigma‐Aldrich, USA) were used to label CD31. All sections were labeled with DAPI (Sigma‐Aldrich, USA) for nuclear staining. Finally, image observation and collection were performed using a fluorescence microscope (Leica Microsystems, Germany) [42].

#### 4.9.2 β‐Galactosidase Staining

Perform staining according to the steps in the referenced literature. First, fix the rat skin tissue and prepare frozen sections. Incubate the sections in SA‐β‐Gal staining buffer (Sigma‐Aldrich, USA) containing X‐gal at 37°C for 12 h. Stop the reaction by washing with PBS, mount with an aqueous mounting medium, and observe under a microscope (Leica Microsystems, Germany) [42].

### 4.10 Statistical Analysis

Statistical analyses for each experiment were conducted in accordance with the instructions provided in the corresponding figure legends, with each experiment performed in *n* = 3 independent replicates. The determination of statistical significance was carried out using GraphPad Prism 9 software. Specifically, one‐way ANOVA, two‐way ANOVA, and Student's t‐tests were applied. A p‐value of less than 0.05 was considered statistically significant and denoted by an asterisk (^*^ *p* < 0.05), while a p‐value greater than 0.05 was regarded as non‐significant and marked as “ns” (ns: p > 0.05).

## Conflicts of Interest

The authors declare no conflicts of interest.

## Supporting information




**Supporting File**: advs73850‐sup‐0001‐SuppMat.docx.

## Data Availability

The data that support the findings of this study are available from the corresponding author upon reasonable request.
