# Supplementary material for: Photothermal–Immunomodulatory Hydrogel Reinforced by Ti3C2/ZnAl‐LDH Nanoplatform for Eradicating MRSA and Promoting Diabetic Wound Healing
Source: Adv Sci (Weinh). 2026 Jan 20;13(17):e17577. doi: 10.1002/advs.202517577 (PMC13042765; doi:10.1002/advs.202517577)
Supplement: Supplementary file 1 — Supporting File: advs73850‐sup‐0001‐SuppMat.docx. [file ADVS-13-e17577-s001.docx]

**Photothermal–Immunomodulatory Hydrogel Reinforced by Ti₃C₂/ZnAl-LDH Nanoplatform for Eradicating MRSA and Promoting Diabetic Wound Healing**

*Qiang Shi ^a, 1^, Renliang Zhao ^c, d^, Jia Wang ^a^, Zhenchen Xiong ^c, d^, Xiangtian Deng ^c, d, *^, Cheng Zheng ^b^, *, Wenzhi Zhang ^a, *^*

^a^ Department of orthopaedics, The First Affiliated Hospital of USTC, Division of Life Sciences and Medicine, University of Science and Technology of China, Hefei, Anhui,230001, China

^b^ National Engineering Research Center for Biomaterials, Sichuan University, Chengdu, 610065, China.

^c^ Orthopedics Research Institute, Department of Orthopedics, West China Hospital, Sichuan University, Chengdu 610041, P.R. China

^d^ Trauma Medical Center, Department of Orthopedics Surgery, West China Hospital, Sichuan University, Chengdu 610041, China.

Corresponding author: E-mail addresses: [350144946@qq.com](mailto:350144946@qq.com) (X. Deng); [zhengcheng@scu.edu.cn](mailto:zhengcheng@scu.edu.cn) (C. Zheng); [wenzhizhang@ustc.edu.cn](mailto:wenzhizhang@ustc.edu.cn) (W. Zhang)

^1^ These authors contributed equally to this work

**Results**


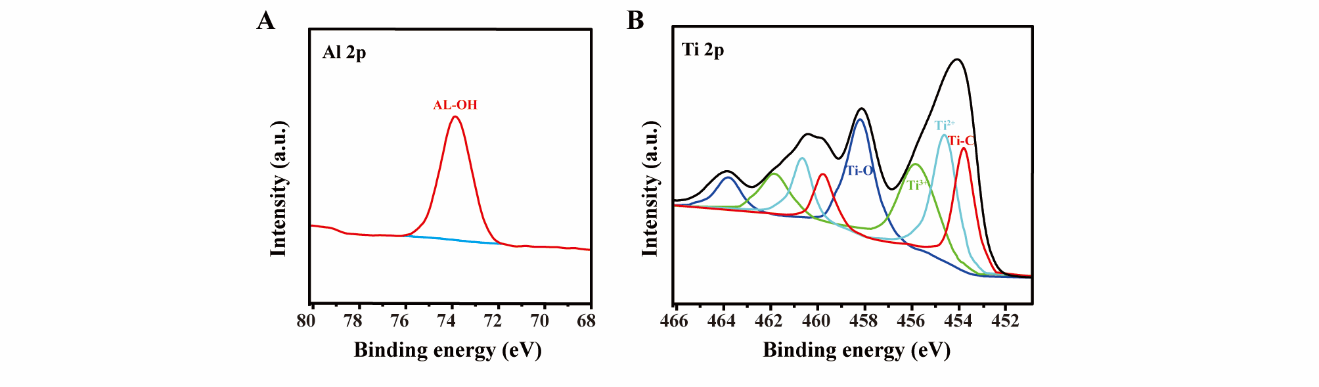


**Figure S1**. (A). XPS analysis of elemental Al; (B). XPS analysis of elemental Ti;


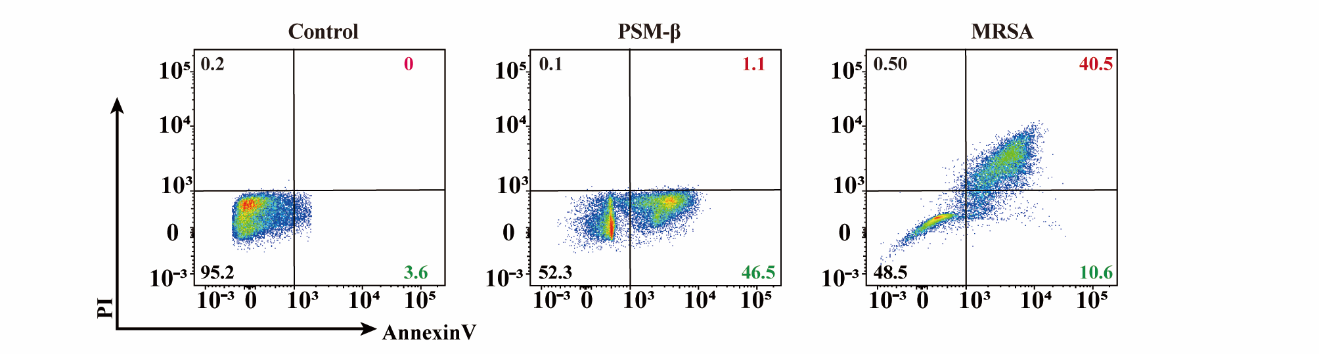


**Figure S2.** Flow cytometric analysis of PSM-β-induced early apoptosis of neutrophils.


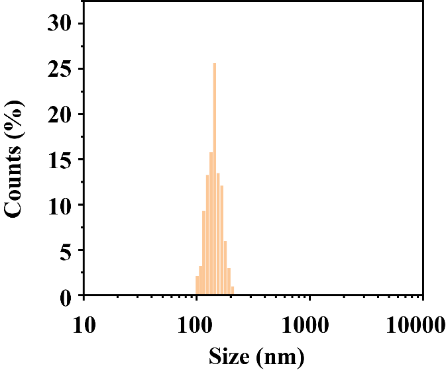


**Figure S3.** Size distribution of the CTiZM

**
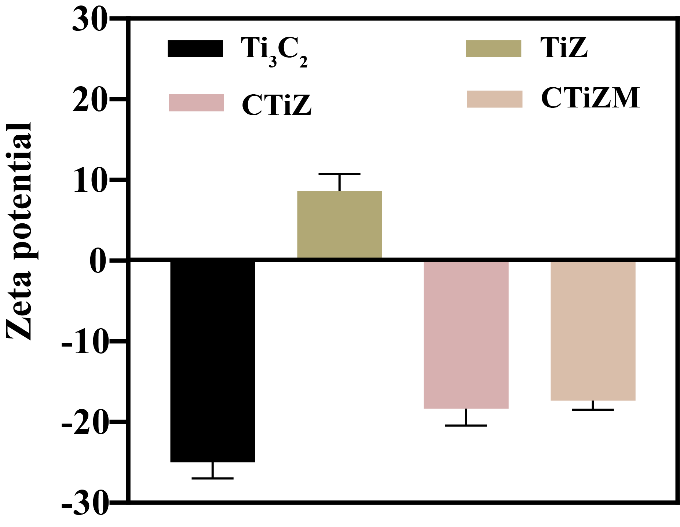
**

**Figure S4**. Zeta potentials of Ti_3_C_2_, TiZ, CTiZ and CTiZM


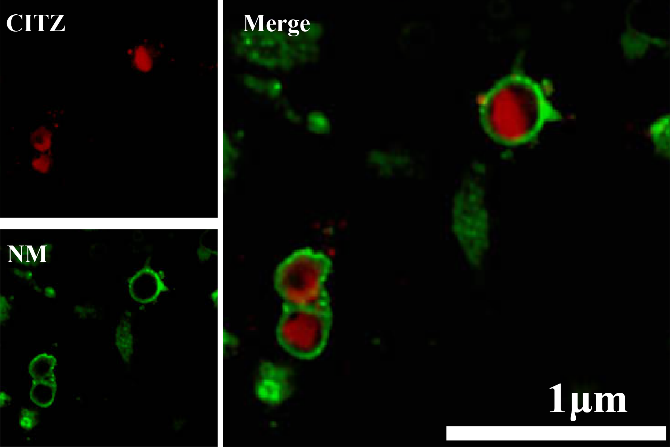


**Figure S5.**  Super-resolution microscopy images showing the co-localization of CTiZM


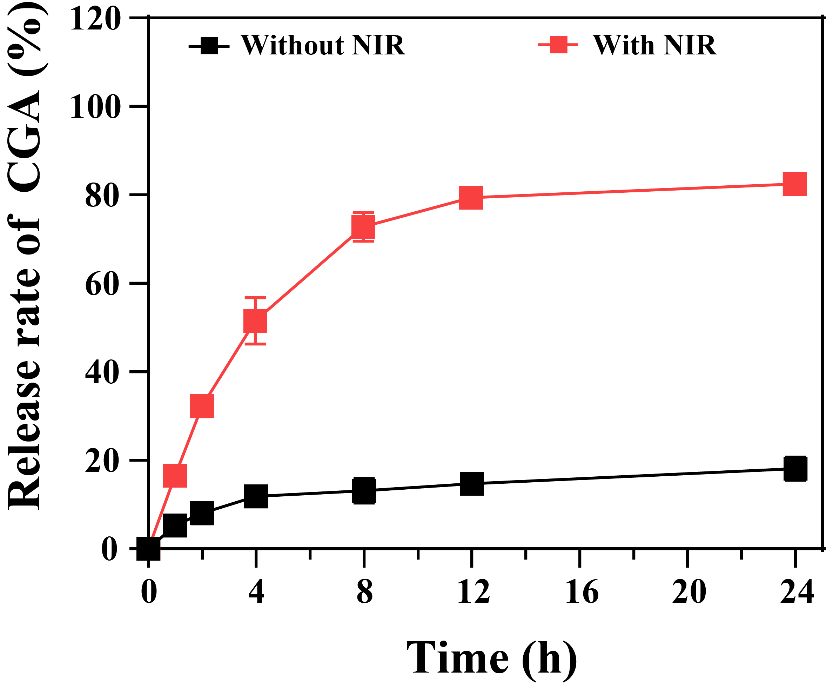


**Figure S6.** In vitro release profile of CGA from the CTiZM nanoparticles under different conditions.

**
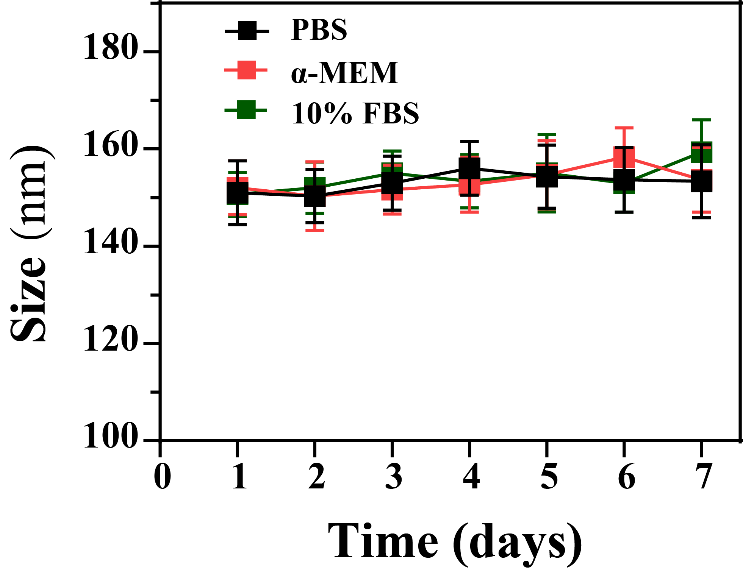
**

**Figure S7.**The [hydrodynamic](https://www.sciencedirect.com/topics/materials-science/hydrodynamics) size changes of CTiZM in various aqueous conditions, including water, α-MEM culture medium with or without 10 % FBS for 7 days.


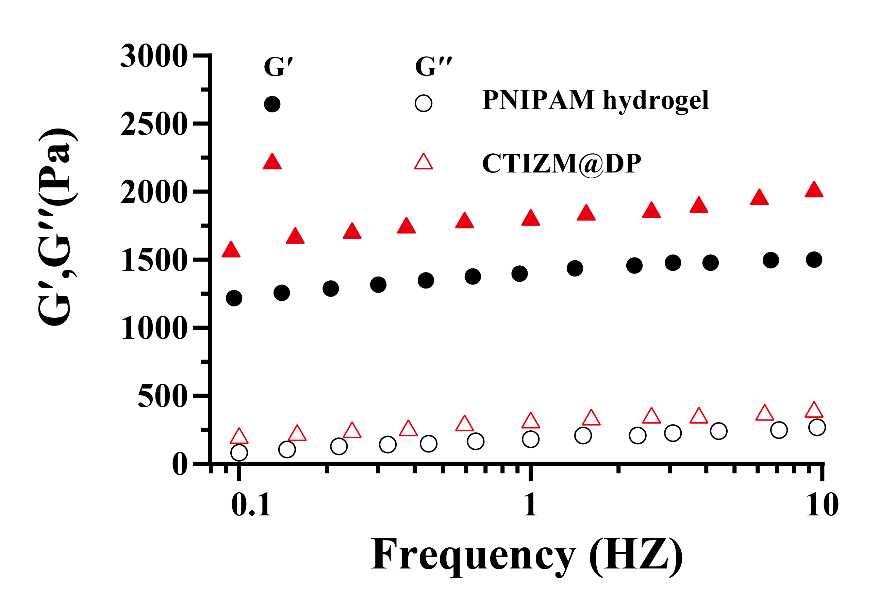


**Figure S8.** Frequency-sweeping rheological behavior of the CTiZM@DP hydrogels


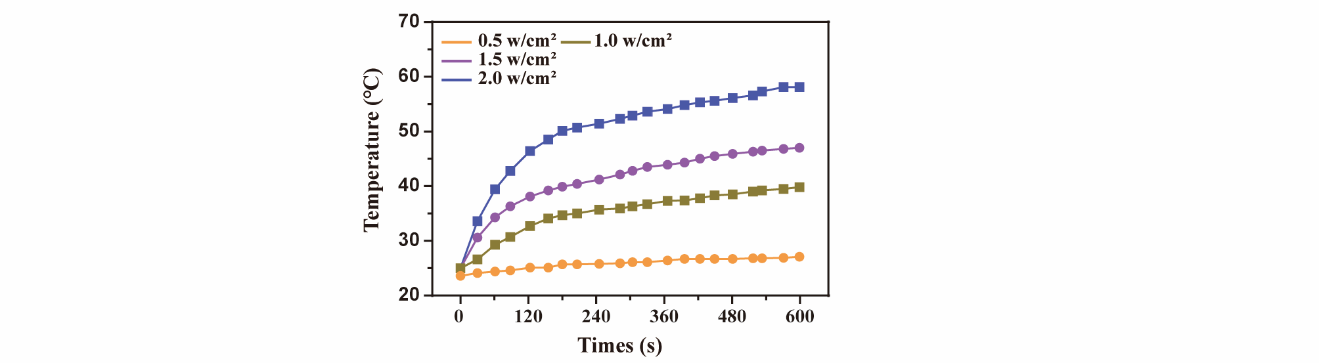


**Figure S9.** In vitro heating curve of the CTiZM@DP hydrogel


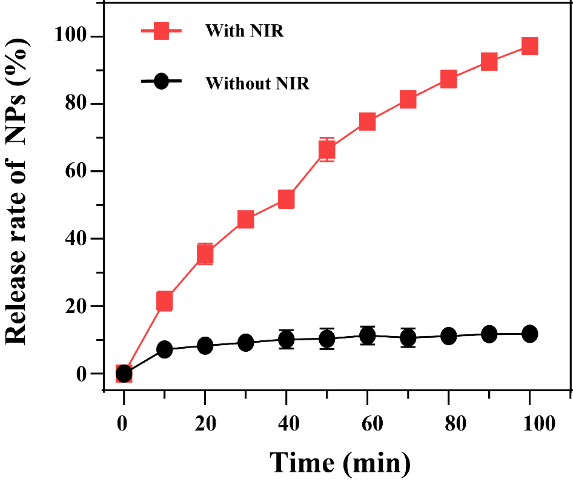


**Figure S10.**The release behavior of the CTiZM@DP hydrogel with or without NIR irradiation.


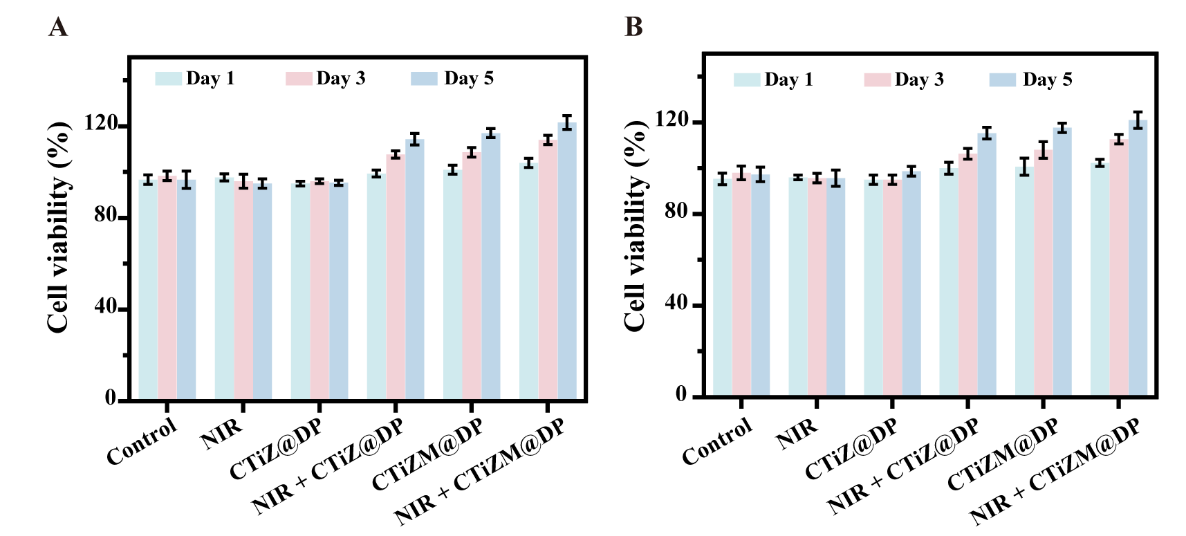


**Figure S11.** Cell viability of 3T3 cells (A) and HUVECs (B) cultured with different hydrogel treatments for 1, 3, and 5 days.


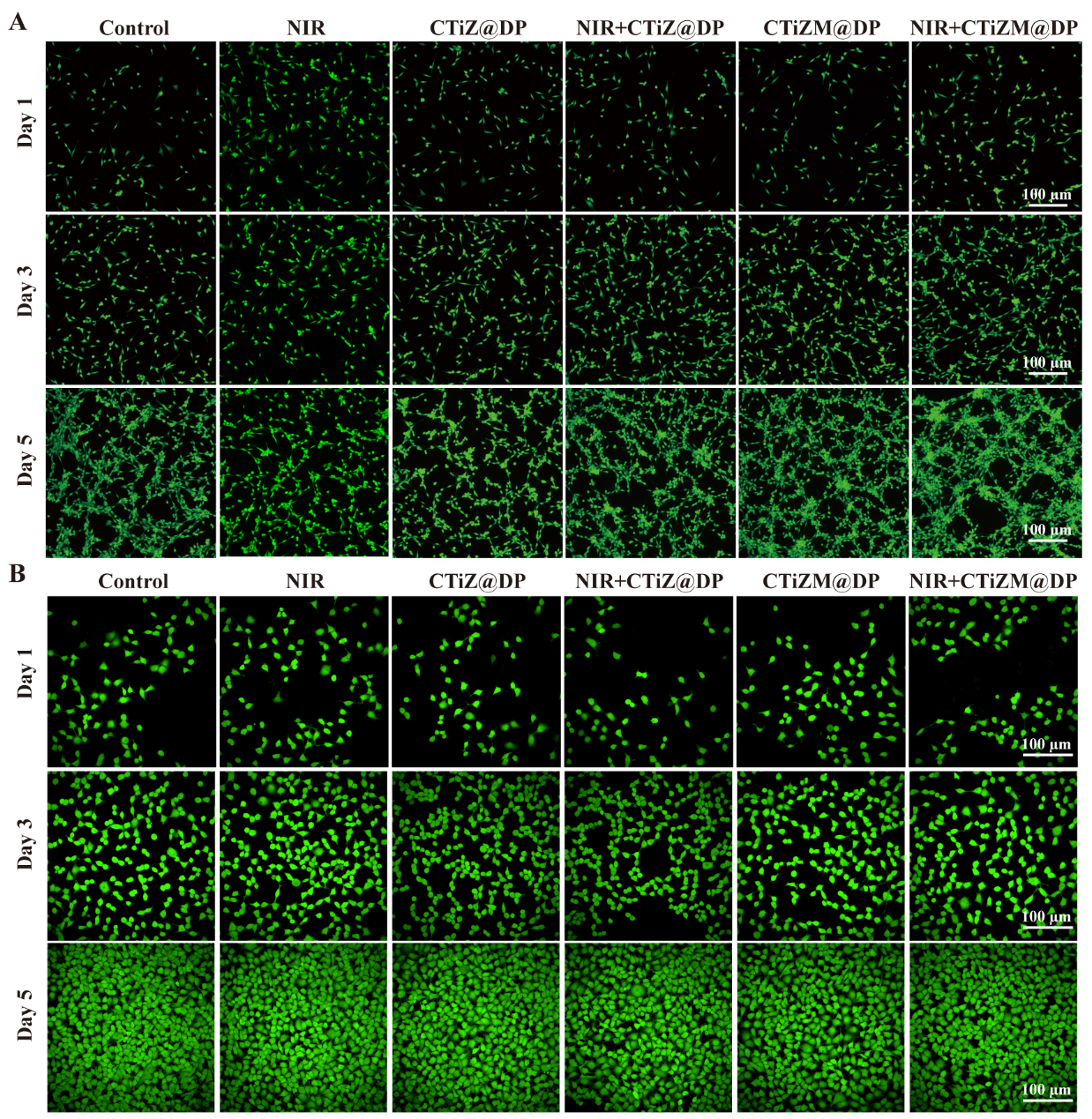


**Figure S12.** Live/Dead fluorescence staining of 3T3-L1 cells (A) and HUVECs (B) cultured with different hydrogel formulations for 1, 3, and 5 days.


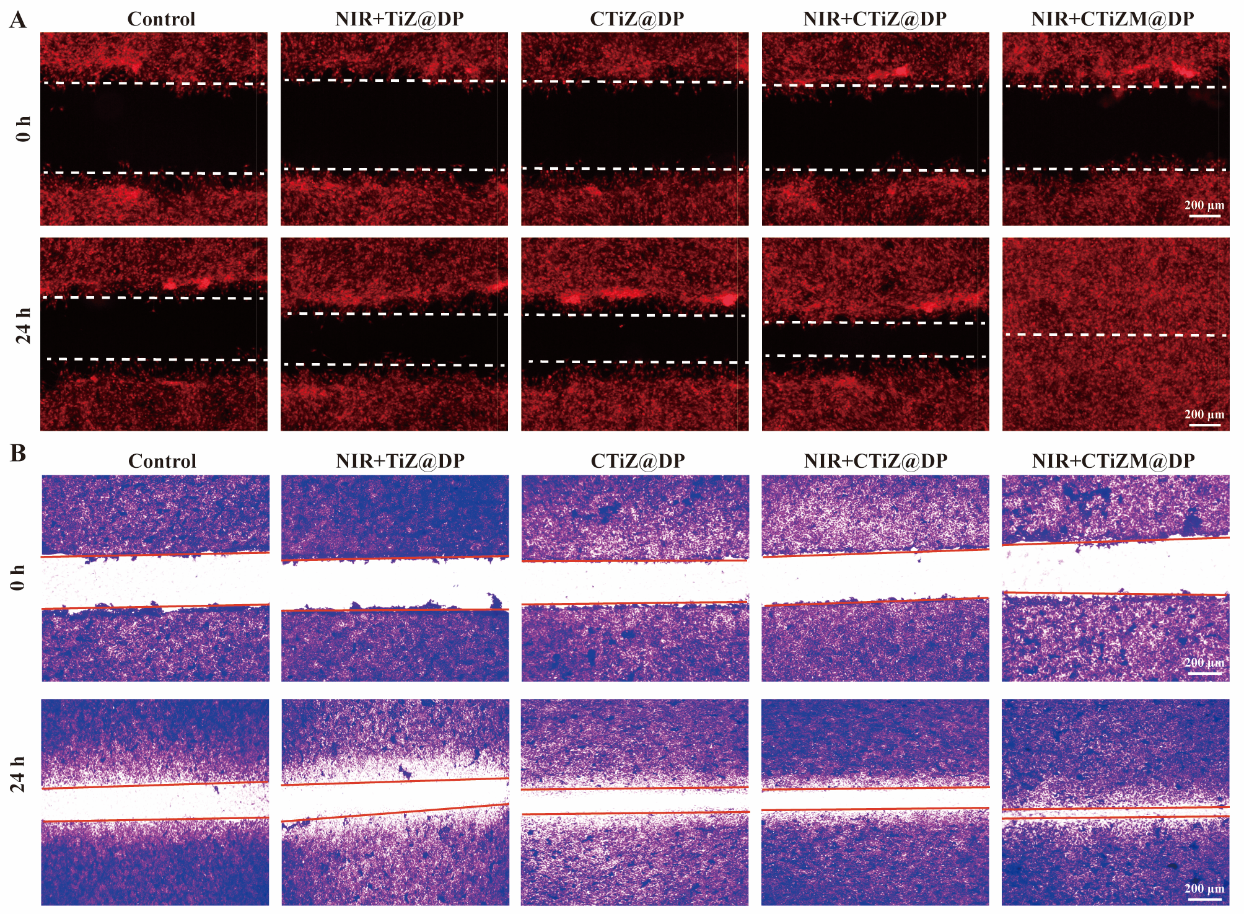


**Figure S13.** Scratch assay images showing the migration of 3T3-L1 cells (A) and HUVECs (B) under different hydrogel treatments at 0 h and 24 h.


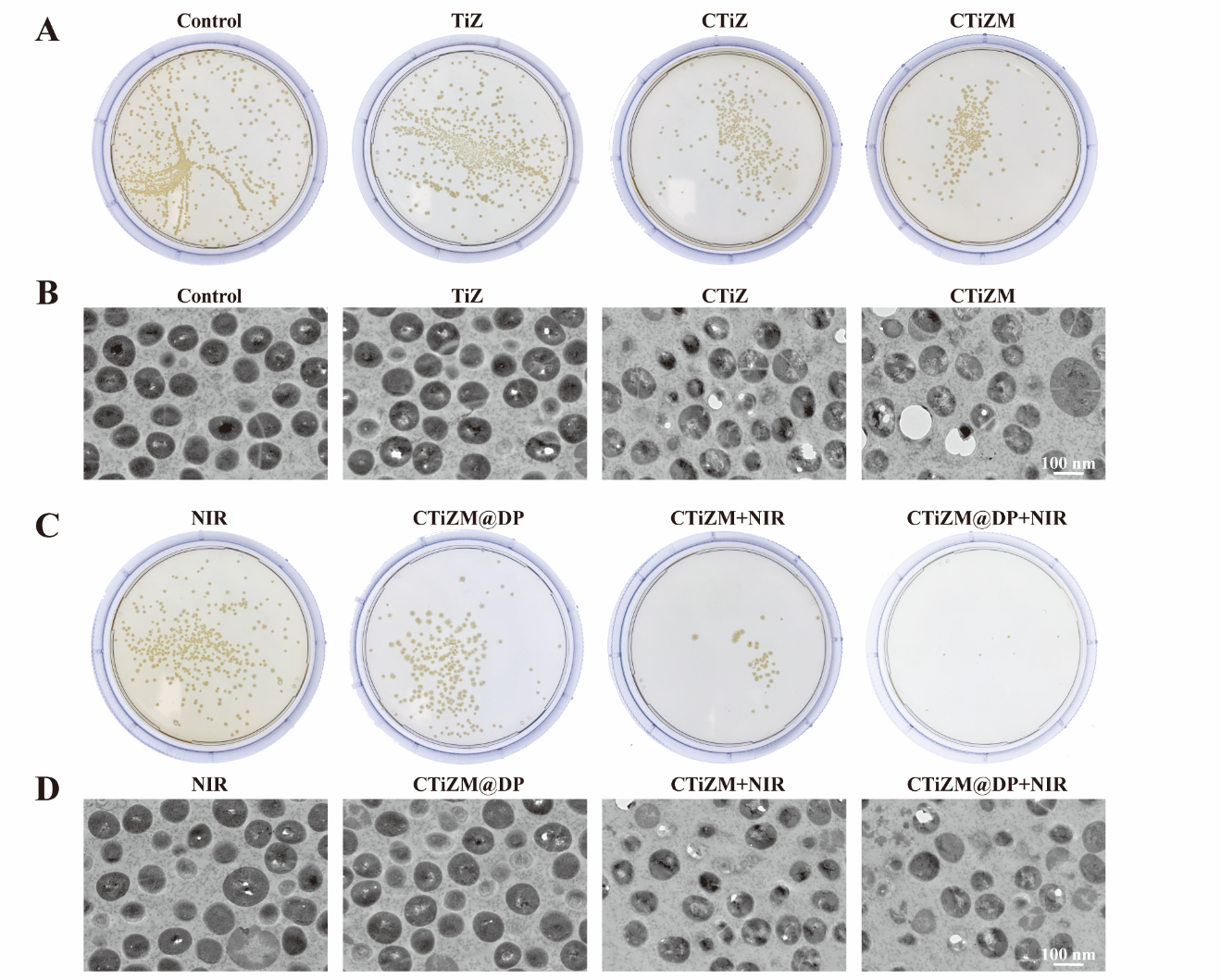


**Figure S14.** In vitro antibacterial properties of CTiZM biomimetic neutrophil extracellular traps and CTiZM@DP composite hydrogel. (A) Colony formation of MRSA after intervention with CTiZM biomimetic neutrophil extracellular traps and (B) analysis of its cell morphology under high magnification; (C) Colony formation of MRSA after intervention with CTiZM@DP composite hydrogel and (D) analysis of its cell morphology under high magnification.


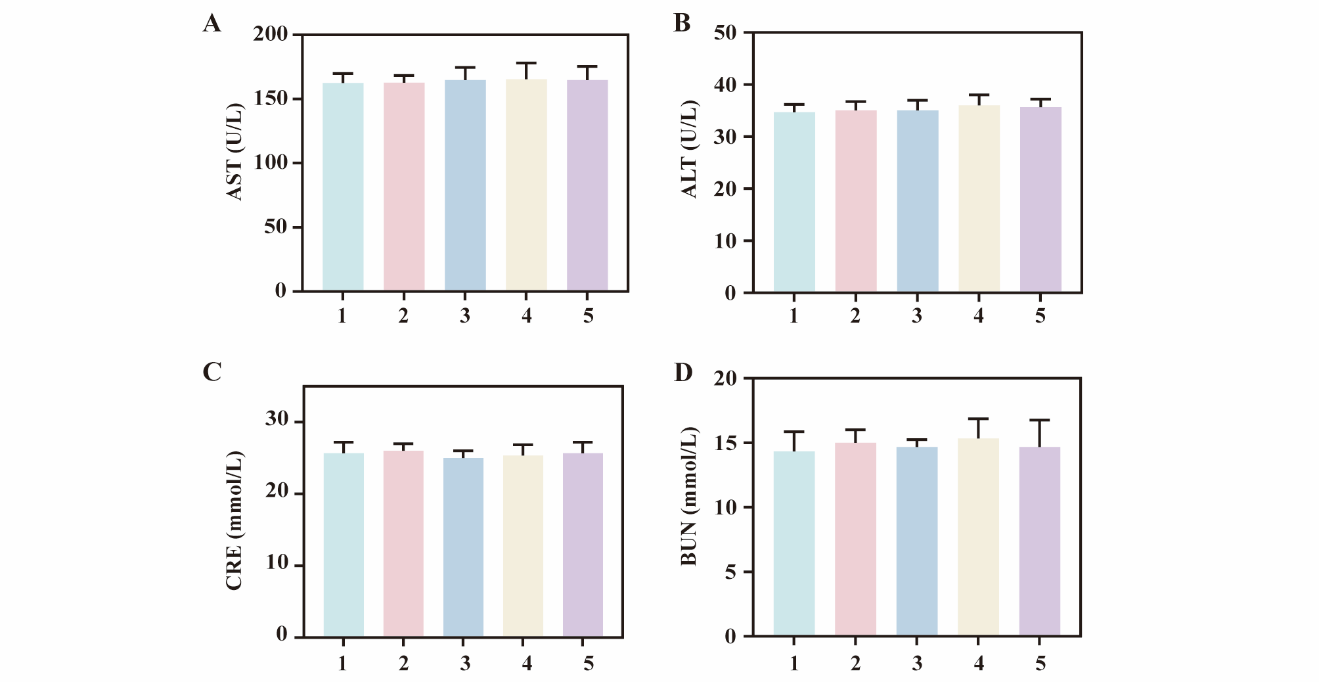


**Figure S15.** In vivo safety assessment at 14 days post-surgery. (A). AST level measurement; (B). ALT level measurement; (C). CRE level evaluation; (D). BUN level measurement (1 represents the Control group, 2 represents the NIR+TiZ@DP group, 3 represents the CTiZ@DP group, 4 represents the NIR+CTiZ@DP group, and 5 represents the NIR+CTiZM@DP group).

**
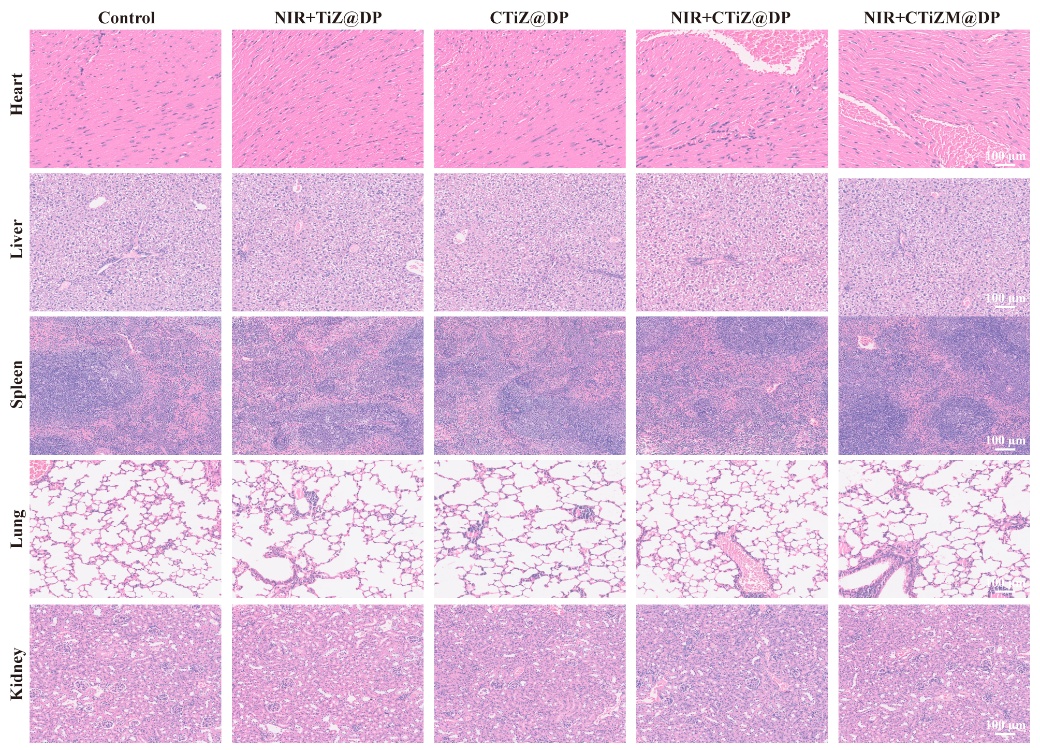
**

**Figure S16.** H&E staining images of organs (heart, liver, spleen, lung, and kidney)


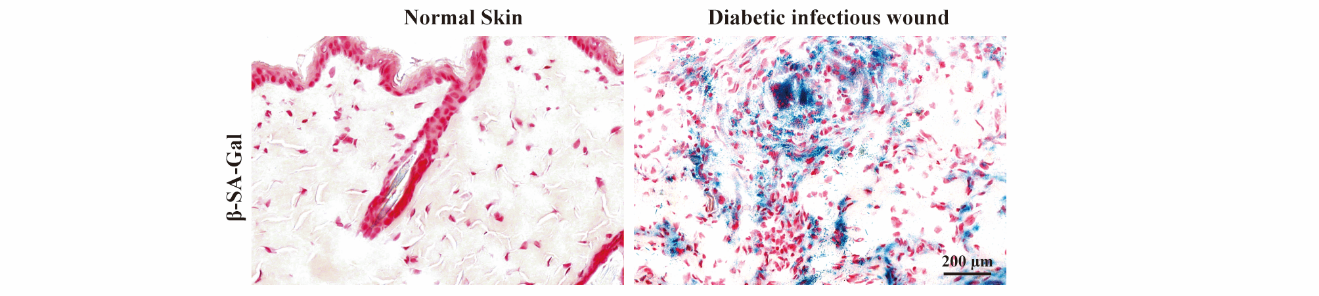
**Figure S17.** Wound tissue β-galactosidase staining.
